# A Single-Center Study of Long-Term Effectiveness of Vedolizumab in Anti-TNF Refractory Pediatric Inflammatory Bowel Disease

**DOI:** 10.1097/PG9.0000000000000276

**Published:** 2022-12-27

**Authors:** Halee Patel, Lina Karam, Richard Kellermayer

**Affiliations:** From the * Department of Pediatrics, Section of Gastroenterology, Hepatology and Nutrition, Baylor College of Medicine/Texas Children’s Hospital, Houston, TX; † Children’s Nutrition and Research Center, Houston, TX.

**Keywords:** vedolizumab, pediatrics, inflammatory bowel disease

## Abstract

Vedolizumab is an anti-α4β7 integrin antibody that has been used successfully in the treatment of adult-onset inflammatory bowel diseases (IBDs: Crohn disease [CD] and ulcerative colitis [UC]). Its off-label use in the pediatric IBD (PIBD) population is increasing, but knowledge on durability beyond 6 months of treatment is limited.

**Methods:**

A real-life, single-center, retrospective study of PIBD patients treated with vedolizumab was performed. Data on demographics, prior and concomitant treatments, and disease activity were obtained at 14 weeks, 26 weeks, 1 year, and 2 years of therapy. Primary outcome was corticosteroid- and other biologic-free remission (based on pediatric ulcerative colitis activity index [PUCAI]).

**Results:**

Thirty-nine patients were studied. By 1 year, 65% of CD and 68% of UC patients continued on vedolizumab therapy. Corticosteroid- and other biologic-free remission was 29% in CD and 16% in UC. By 2 years, 36% of CD and 47% of UC patients continued therapy. Corticosteroid- and other biologic-free remission was 21% in CD and 40% in UC. By 2 years, 80% of CD and 100% of UC patients were on intensified treatment regimen compared to the manufacturer guidance. Nine patients (23%) required surgical intervention within 26 months of starting vedolizumab indicating the severity of IBD in this cohort.

**Conclusions:**

Vedolizumab is a useful therapeutic modality in PIBD patients refractory to anti-TNF therapy, although with declining effectiveness by 2 years. Intensified treatment regimens are associated with long-term durability. Larger prospective trials in children are warranted.

**What Is Known**

- Several pediatric studies have shown that vedolizumab may be safe and effective in anti-TNF refractory cases of inflammatory bowel disease (IBD).
- The effectiveness of vedolizumab has been studied in the adult population, but there is limited data on its long-term use and durability in children.

**What Is New**

- Long-term efficacy of vedolizumab declines over 2 years in pediatric IBD (PIBD) patients.
- Intensified dosing of vedolizumab compared to the standard adult regimen is progressively needed to maintain therapeutic efficacy.
- Our findings further support the concept that biologic pharmacokinetics may differ between adult and pediatric IBD patients.

## INTRODUCTION

A continuing rise of pediatric inflammatory bowel disease (PIBD) incidence has been observed in several recent studies (1–3). PIBD is frequently more aggressive than the adult onset and even the highly efficient anti-tumor necrosis factor-alpha (anti-TNF) biologic agents can fail primarily or over time (ie, secondary failure) (4–6). Therefore, novel and optimized modes of treatment are critically needed in these patients, especially in those with anti-TNF therapy failure.

Vedolizumab is an anti-α4β7 integrin antibody with gut-selective anti-inflammatory activity that has been used successfully in the treatment of adult-onset IBDs (Crohn disease [CD] and ulcerative colitis [UC]) (7). Vedolizumab acts on the α4β7 integrin receptor on lymphocytes, blocking their interaction with MadCAM-1 on the intestinal endothelium, and thereby inhibiting lymphocyte migration to the intestinal mucosa. As this interaction is gut-selective, the risks of systemic immunosuppression that were seen with the drug’s predecessor (natalizumab) on the central nervous system are significantly decreased (8).

The clinical trials of GEMINI 1, 2, and 3 demonstrated the durability of vedolizumab in adult patients, notably with better results in UC compared to CD (8–10). More recently, the Cross Pennine study in adults demonstrated the long-term effectiveness and appropriate safety profile of vedolizumab; 78.5% of CD patients and 91.2% of UC patients showed clinical response or remission at 14 weeks, and 63.9% of CD and 82.5% of UC patients continued to show response or remission at 52 weeks (11).

The off-label use of vedolizumab and data on its efficacy in PIBD are increasing. Singh et al (8) in 2016 reported that at week 14, 42% of CD patients, and 76% of UC patients were in clinical remission (n = 52). This study also found improved remission rates for anti-TNF-naive patients in contrast to patients with previous exposure of anti-TNF agents (100% n = 4 versus 45% n = 28, *P* = 0.04). Safety of vedolizumab was also indicated in this work. Several other pediatric studies demonstrated that vedolizumab is safe and effective for use in PIBD up to week 22 (12,13) and week 38 (14) of treatment. Hajjat et al (15) published a multicenter retrospective study in 2021 in which 43% of pediatric patients were observed to achieve corticosteroid free remission on vedolizumab at 1 year. Additionally, data from the phase 2 HUBBLE study revealed that vedolizumab serum concentrations increased in a dose-proportional manner, although no clear dose–response relationship was observed (16). This study was limited in its sample size, but was the first to report pharmacokinetic data for vedolizumab use in children. Thus, data are limited regarding the effectiveness of vedolizumab beyond 6 months of treatment. We aimed to examine the long-term efficacy of vedolizumab therapy in our pediatric population at a tertiary PIBD center.

## MATERIALS AND METHODS

Pediatric patients who were initiated on vedolizumab at Texas Children’s Hospital in Houston, TX between September 2015 and September 2018 and completed the induction phase of treatment (through week 14) were included in this study. The decision to initiate vedolizumab was at the discretion of the treating physician. Pertinent data were collected through the end of the study period in September 2020 if available. The study was approved by the Institutional Review Board of Texas Children’s Hospital, Baylor College of Medicine (H-43380).

Age at diagnosis, age at vedolizumab initiation, previous or concomitant corticosteroid, biological or immunomodulatory therapy, disease activity, and surgical history were collected. Disease activity was defined by the Pediatric Ulcerative Colitis Activity Index (PUCAI) and retrospectively calculated by chart review for all patients, including patients with CD. Due to the retrospective nature of this study, several data required for calculating the Pediatric Crohn’s Disease Activity Index (PCDAI) and even the abbreviated PCDAI were unavailable. Therefore, we decided to calculate PUCAI scores for all patients, including those with CD, as the primary burden of their disease was colonic/ileocolonic, as has been done in prior studies (17). Data on laboratory biomarkers or endoscopic evaluations were not routinely available at the required timepoints and therefore were not evaluated in this study. Data on disease activity specifically were focused at 14 weeks, 26 weeks, 1 year, and 2 years of therapy. Dosing regimens including the frequency of infusions were recorded. Vedolizumab drug levels and timing of therapeutic drug monitoring (TDM) were documented if available. Data on adverse events were also collected.

The primary outcome of the study was defined as corticosteroid- and other biologic agent-free remission at 26 weeks and 1 year. Clinical remission was defined as a PUCAI score of less than 10. Mild disease activity was defined as a PUCAI of 10–34 and moderate/severe disease activity was defined as a PUCAI > 35 (18). Secondary outcomes included discontinuation of therapy, corticosteroid- and other biologic agent-free remission at 14 weeks and 2 years of treatment, need for surgical intervention, and the time from initiation of vedolizumab to surgical intervention.

Data were reported in percentage of patients achieving remission and were compared across independent groups by using the Fischer exact test. The statistical significance level was set at *P* < 0.05.

## RESULTS

### Patient Characteristics

A total of 39 patients completed the initial induction treatment of vedolizumab for CD (49%) or UC (51%). Data on baseline characteristics are reported in Table 1. The predominant CD phenotype was L3/B1 (ileocolonic [63%], nonstricturing, nonpenetrating) according to Paris classification (19). The predominant UC phenotype was pancolitis (E4, 95%) with 100% having an episode of ever having severe disease (PUCAI score > 65). The mean age at initiation of vedolizumab was 14.5 years with an age range of 5–19. Thirty-eight of the 39 patients (97%) were refractory to previous anti-TNF therapy (defined as having an inadequate response to the agent as primary nonresponse, or secondary loss of response, or adverse reaction). Only 1 of 39 (3%) patient with CD (L2) was anti-TNF-naive, who was initiated on vedolizumab after strict specific carbohydrate diet and oral vancomycin therapy failed. Overall, 16 (41%) of the patients had been treated with a second anti-TNF agent (adalimumab) before vedolizumab.

**TABLE 1.** Baseline patient characteristics at start of vedolizumab therapy

	Total [n = 39]	CD [n = 19]	UC [n = 20]
Male	19 [49%]	9 [47%]	10 [50%]
Ethnicity			
Hispanic	9 [23%]	3 [16%]	6 [30%]
Non-Hispanic	30 [77%]	16 [84%]	14 [70%]
Age at vedolizumab initiation, y, mean (range)	14.5 [5–19]	14.3 [6–19]	14.8 [5–19]
Disease duration, mo, mean (range)	52.3 [3–201]	70.2 [10–201]	35.4 [3–143]
No. of previous biologic agents, n (%)			
0	1 [3%]	1 [5%]	0 [0%]
1	22 [56%]	9 [47%]	13 [65%]
2	16 [41%]	9 [47%]	7 [35%]
Previous biologic agents, n (%)			
Infliximab	33 [85%]	13 [68%]	20 [100%]
Adalimumab	19 [49%]	12 [63%]	7 [35%]
Certolizumab	1 [3%]	1 [5%]	0 [0%]
Ustekinumab	1 [3%]	1 [5%]	0 [0%]
Reason for discontinuation, n (%)			
Infliximab			
Primary nonresponder	11 [33%]	2 [15%]	9 [45%]
Loss of response	12 [36%]	6 [46%]	6 [30%]
Adverse reaction	9 [27%]	4 [31%]	5 [25%]
Other	1 [3%]	1 [8%]	0 [0%]
Adalimumab			
Primary nonresponder	4 [21%]	1 [8%]	3 [43%]
Loss of response	15 [79%]	11 [92%]	4 [57%]
Certolizumab			
Loss of response	1 [100%]	1 [100%]	0 [0%]
Ustekinumab			
Primary nonresponder	1 [100%]	1 [100%]	0 [0%]
Behavior phenotype (CD), n (%)			
Nonstricturing, nonpenetrating [B1]	–	12 [63%]	–
Stricturing [B2]	–	1 [5%]	–
Penetrating [B3] Both structuring and penetrating [B2B3]	– –	1 [5%] 5 [26%]	– –
Lower gastrointestinal involvement (CD), n (%)			
Terminal ileum only [L1]	–	0 [0%]	–
Colonic only [L2]	–	7 [37%]	–
Ileocolonic [L3]	–	12 [63%]	–
Upper gastrointestinal involvement (CD), n (%)	–	15 [79%]	–
Perianal involvement (CD), n (%)	–	7 [37%]	–
Behavior phenotype (UC), n (%)			
Ulcerative proctitis [E1]	–	–	0 [0%]
Left-sided UC [E2]	–	–	0 [0%]
Extensive [E3]	–	–	1 [5%]
Pancolitis [E4]	–	–	19 [95%]
Severity (UC), n (%)			
Never severe [S0]	–	–	0 [0%]
Ever severe [S1]	–	–	20 [100%]

CD = Crohn disease; UC = ulcerative colitis.

### Details of Vedolizumab Induction

Of the 39 patients who completed the initial three dose induction of vedolizumab, 20 (51%) received the “standard” induction regimen (ie, according to manufacturer recommendation) with vedolizumab infusions administered at 0, 2, 6, and 14 weeks. The remaining 19 patients underwent a modified induction at their primary gastroenterologist’s discretion in response to either persistent or worsening symptoms (reactive change in infusion schedules), or prospectively, based on subjective clinical experience. Fourteen of these patients had interval change compared to the standard after the third (week 6) infusion and the remaining 5 patients had intensification before week 6 of therapy.

Combination treatment regimens during induction varied among the patients; 7 patients with CD (37%) and 9 patients with UC (45%) were given corticosteroids alone for induction but 16% of patients with CD and 25% of patients with UC were concomitantly on another agent such as a biologic or immunomodulator in addition to the corticosteroids. Dual biologic therapy was used in 8 patients with CD (42%) which included 6 patients on adalimumab, 1 patient on infliximab, and 1 patient on ustekinumab. Dual biologic therapy was used in 5 patients with UC (25%) during induction, which included 3 patients on adalimumab and 2 patients on infliximab. These patients remained on their prior biologic agent as bridge therapy while undergoing induction with vedolizumab at the discretion of their primary gastroenterologist. Data for induction regimens are summarized in Supplemental Digital Table 1, http://links.lww.com/PG9/A98.

Thirty-three (85%) patients received the standard, adult dose of vedolizumab (300 mg) and the remaining 6 patients received ~6 mg/kg dose (ranging from 100 to 200 mg per dose). The youngest patient to receive the 300 mg dose was 9 years old at the start of vedolizumab.

### Clinical Remission on Vedolizumab

**FIGURE 1.**
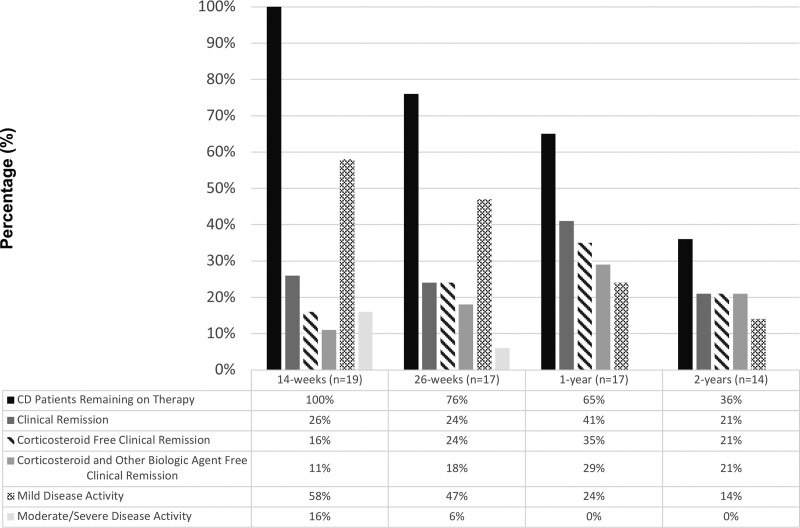
Disease activity and clinical outcomes in patients with CD on vedolizumab treatment at 14 weeks, 26 weeks, 1 year, and 2 years. CD = Crohn disease.

**FIGURE 2.**
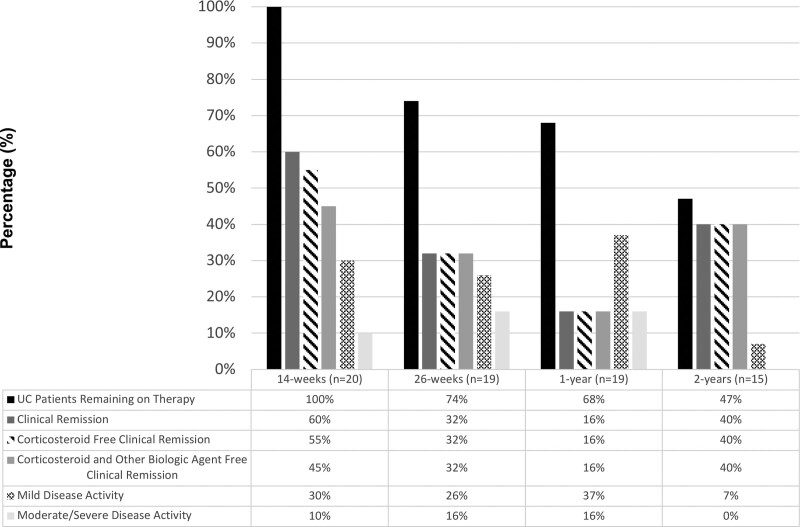
Disease activity and clinical outcomes in patients with UC on vedolizumab treatment at 14 weeks, 26 weeks, 1 year, and 2 years. UC = ulcerative colitis.

Data were available on 36 of 39 patients (92%) at 26 weeks and 1 year after the initiation of vedolizumab (Supplemental Digital Figure 1, http://links.lww.com/PG9/A98). Two-year data were available for 29 of 39 patients (74%). At week 14, 26% (5/19) of CD and 60% (12/20) of UC patients achieved clinical remission and 11% (2/19) of CD and 45% (9/20) of UC patients achieved both corticosteroid- and other biologic-free remission (Figs. 1 and 2). At week 26, 24% (4/17) of CD and 32% (6/19) of UC patients achieved clinical remission and 18% (3/17) of CD and 32% (6/19) of UC patients achieved corticosteroid- and other biologic-free remission, respectively. At 1 year, 29% (5/17) of CD and 16% (3/19) of UC patients achieved corticosteroid- and other biologic-free remission. At 2 years, 21% (3/14) of CD and 40% (6/15) of UC patients had achieved clinical remission without requiring any corticosteroid or other biologic agents. At 2 years, only 4 (2 CD, 2 UC) patients were receiving vedolizumab monotherapy (including no immunomodulator or salicylate therapy).

### Details on Dual Biologic Use

Patients who received dual biologic therapy (vedolizumab plus another biologic agent) at 14 weeks, 26 weeks, or 1 year did not demonstrate a significant difference in clinical remission or corticosteroid-free remission rates (*P* > 0.1) compared with patients on vedolizumab monotherapy. However, IBD patients not on dual biologic agents at 2 years were more likely to be in corticosteroid-free clinical remission compared with IBD patients requiring vedolizumab plus another biologic agent (*P* = 0.004). Combination regimens are further described in Table 2.

**TABLE 2.** Combination therapeutic agents applied with vedolizumab in the patients who were maintained on this biologic

	**14 Wks**	**26 Wks**	**1 Y**	**2 Y**
CD	n = 19	n = 13	n = 11	n = 5
Corticosteroids	8 [42%]	2 [15%]	2 [18%]	0 [0%]
Other biologic agents	7 [37%]	4 [31%]	4 [36%]	2 [40%]
Other immunomodulators	4 [21%]	3 [23%]	3 [27%]	1 [20%]
UC	n = 20	n = 14	n = 13	n = 7
Corticosteroids	5 [25%]	2 [14%]	0 [0%]	0 [0%]
Other biologic agents	3 [15%]	2 [14%]	2 [15%]	1 [14%]
Other immunomodulators	7 [35%]	4 [29%]	3 [23%]	2 [29%]

CD = Crohn disease; UC = ulcerative colitis.

Of the 8 patients with CD who continued to receive another biologic agent during the induction of vedolizumab, 13% (1/8) achieved clinical remission at week 14, but 75% (6/8) continued to have mild disease activity. The 1 patient who achieved clinical remission at week 14 was on ustekinumab concomitantly during induction and maintenance, but ultimately, was taken off vedolizumab before reaching the 1-year timepoint. Furthermore, of the mild disease activity group, 1 patient ultimately achieved clinical remission by 26 weeks and remained on vedolizumab monotherapy by 1 year and 2 years post vedolizumab initiation.

Of the 5 patients with UC who continued to receive an anti-TNF agent during the induction of vedolizumab, 60% (3/5) achieved corticosteroid free clinical remission at week 14. Two of the patients were on adalimumab during induction and remained on adalimumab through 26 weeks and 1 year but thereafter developed mild disease activity despite the dual biologic therapy. Furthermore, the remaining patient who had achieved corticosteroid-free clinical remission at week 14 was on infliximab during induction but was taken off of infliximab at week 14. This patient continued to remain in corticosteroid-free clinical remission at the 2-year timepoint on vedolizumab monotherapy.

A second biologic agent was not added for any patients with UC who were initiated on vedolizumab monotherapy. However, 3 patients with CD who had undergone standard induction with vedolizumab alone required the addition of a second biologic agent at or after 1 year of the monotherapy. One of these patients received infliximab but ultimately stopped vedolizumab therapy. The other 2 patients received ustekinumab in addition to vedolizumab and ultimately only one of the 2 patients was able to remain on dual ustekinumab/vedolizumab therapy at 2 years but with continued mild disease activity.

Corticosteroid free remission rates in patients with colonic only disease (including UC and colonic only CD) was no different compared with patients with SB involvement (ileocolonic CD) at both 26-week (colonic only 50% versus ileocolonic 50%) and 1-year (colonic only 58% versus ileocolonic 50%) timepoints. Clinical remission or corticosteroid plus other biologic agent free remission rates also did not differ.

No significant (*P* > 0.1) difference was found relating to gender in any of the outcomes examined.

### Durability of Vedolizumab

At week 26, 76% (13/17) of patients with CD and 74% (14/19) with UC remained on therapy (Figs. 1 and 2). Of the patients with 52-week outcomes available, 65% (11/17) with CD and 68% (13/19) with UC remained on vedolizumab. By 2 years, 9 patients with CD and 8 patients with UC had discontinued vedolizumab therapy due to severity of disease. No significant (*P* > 0.05) difference between CD or UC was found in any of these outcomes.

Among the patients remaining on vedolizumab at 1 year, 64% of CD patients (4-week interval n = 3, 6-week interval n = 4) and 85% of UC patients (4-week interval n = 6, 6-week interval n = 5) were on an intensified regimen compared with the adult conventional dosing of every 8-week infusions (Supplemental Digital Figure 2, http://links.lww.com/PG9/A98). This ratio of intensified treatment increased to 80% of CD (4-week n = 3, 6-week n = 1) and 100% of UC patients (4-week n = 5, 6-week n = 2) by 2 years. No significant (*P* > 0.05) difference between standard versus intensified dosing regimens was observed in any of these outcomes.

### Surgical Outcomes

Seven patients with UC (35%) and 2 patients with CD (10%) required surgical intervention (partial/total colectomy or diverting ileostomy) following the initiation of vedolizumab. The time from initiation of vedolizumab to surgical intervention varied from 3 to 26 months (median time of 14 months). Among all patients who required surgical intervention, 3 patients were in clinical remission by week 14, of which 2 patients also met criteria for corticosteroid free remission. However, by 1 year, only 2 patients remained in corticosteroid-free remission. Four patients required surgical intervention in <1 year from the start of vedolizumab. All 4 of these patients were noted to have mild to moderate disease activity at week 14 and 75% (3/4) of the patients subsequently had discontinued vedolizumab before reaching week 26. The remaining patient had undergone a diverting ileostomy at 12 weeks following the initiation of vedolizumab but was able to enter clinical remission by 26 weeks and continued to remain in corticosteroid-free remission at the 1-year timepoint.

### Therapeutic Drug Monitoring

Twenty 20 patients (51%) (9 CD and 11 UC) had therapeutic drug monitoring (TDM: vedolizumab levels and antibodies). TDM timing was not standardized and the decision to obtain vedolizumab levels and antibodies was directed by the individual physicians. None of these patients developed antibodies to vedolizumab. Meaningful analyses could not be performed on vedolizumab TDM in this cohort due to the inconsistency in the timing.

### Patient Safety

No serious adverse reactions to vedolizumab during the observation period were reported. One mild, possible drug related event was reported in a patient who developed nausea and vomiting immediately following the fourth dose and was thereby discontinued from further therapy of vedolizumab.

## DISCUSSION

We report the largest real-life PIBD cohort treated with vedolizumab with 1- and 2-year outcomes to date. Response to vedolizumab or the effectiveness of this biologic decreased over time. Other pediatric studies generally report similar findings, but have not examined 2-year outcomes (8,12–15). In the 2017 study by Ledder et al, at week 14, 25% of CD patients (n = 16) and 47% of UC patients (n = 34) were in corticosteroid free clinical remission. At week 22, 36% of CD (n = 14) and 46% of UC patients (n = 26) were in clinical remission. This study also reported 1-year data with 25% (1/4) of CD patients and 60% (6/10) of UC patients in clinical remission and, ultimately, noted that vedolizumab was effective, especially in UC, in inducing and maintaining remission during long-term use (13). UC-specific efficacy of vedolizumab did not reach statistical significance compared with CD (*P* = 0.56). Our study supports this latter result, since no difference in outcomes between patients with CD and UC patients was observed. This finding underscores the importance of independent, larger cohort examinations of biologic effectiveness in PIBD. Our results also reflect adult trial outcomes, which report 32% of CD patients and 39% of UC patients in corticosteroid free clinical remission at 1 year, although only 16% of our UC patients were in clinical remission at the same time point (7). Our cohort also included several patients who received dual biologic therapy from the initiation or during the maintenance phases. Noteworthy, patients who were on dual biologic agents at 2 years were less likely to be in clinical remission compared with patients on single biologic therapy (*P* = 0.004). This alludes to the disease severity of our cohort. As reported by others, we did not find any serious adverse reaction to vedolizumab.

Most of our patients who were maintained on vedolizumab past 1- and 2-year timepoints required interval intensification. All patients with UC at 2 years were on an intensified regimen. These findings suggest that intensified regimens may support the long-term maintenance of vedolizumab therapy. Intensified therapy of biologic agents in PIBD is becoming increasingly more common compared with standard adult practices (18). Jongsma et al recently reported that younger pediatric patients on infliximab were more likely to be on intensified therapy to maintain clinical remission at 1 year (20). Similar results have been observed for other biologics such as adalimumab (21) and ustekinumab (22) in the pediatric population. Our study also favors the use of intensified therapy for maintenance of vedolizumab in PIBD.

With increasing availability and ease of testing, TDM for other biologic agents such as adalimumab and infliximab in pediatric patients is becoming more common to optimize treatment and potentially clinical outcomes (23). TDM of vedolizumab has been indicated as a useful tool in adult patients (24). However, in the pediatric population, data on TDM for vedolizumab are limited to 1 study recently published by Aardoom et al who concluded that patients with CD may benefit from routine TDM and intensified dosing regimens (25). The limited data on vedolizumab TDM in the pediatric population are likely due to the lack of standardized level testing in clinical practice. Prospective studies on pediatric pharmacokinetics for vedolizumab such as reported recently by Hyams et al will further our understanding on the optimized use of vedolizumab and TDM in PIBD (16).

Although the regional/single-center nature of this work may be considered a limitation, Shiau et al (26) have suggested that consistency of medical care in single centers may improve the accuracy of clinical studies in IBD. A recent study from the largest prospective cohort on PIBD patients has underscored the significant variation in clinical care (including diagnosis and treatment) among the North American medical centers involved (27). This work supports our premise on single-center studies potentially providing higher accuracy when examining questions on management of PIBD even with smaller sample sizes than in multicenter cohorts. Regardless, our single-center study also calls for standardized approaches with respect to TDM (by highlighting the lack thereof in real-life practice at a single center).

This study is limited by its retrospective nature and therefore in the ability to control for treatment regimens and for testing and follow-up. PUCAI scores were used in all patients, including those with CD, due to limited data available and inability to calculate CD-specific scores for disease activity (PCDAI). Although the largest single-center cohort of its kind, this work is limited by its cohort size and the lack of standardized measures for TDM use as highlighted above. Furthermore, our study included CD patients with only ileocolonic or colonic predominant disease. Therefore, our data cannot be extrapolated for CD patients with small bowel only or upper gastrointestinal disease.

Our observations indicate that vedolizumab is safe, but its overall efficacy declines with time in anti-TNF exposed CD and UC patients. Our findings also favor the need for intensified treatment regimens of vedolizumab in PIBD to promote long-term maintenance of therapy. These findings emphasize the need for prospective optimization of treatment with vedolizumab and the ongoing requirement for novel preventative and therapeutic measures to combat this highly morbid disease group.

## ACKNOWLEDGMENTS

R.K. was supported in part by philanthropic funds from the Brock Wagner Family led Gutsy Kids Fund including the Frugoni family and other generous donors, and by the Klaasmeyer family funds for PSC research, as well as the ProKIIDS Network of the Crohn’s and Colitis Foundation. H.P. collected and analyzed data, wrote manuscript draft; L.K. provided critical review of the manuscript and contributed to the final submission; R.K. performed conceptual design, data analysis, and manuscript writing.

## Supplementary Material
