# Supplementary material for: A Single-Center Study of Long-Term Effectiveness of Vedolizumab in Anti-TNF Refractory Pediatric Inflammatory Bowel Disease
Source: JPGN Rep. 2022 Dec 27;4(1):e276. doi: 10.1097/PG9.0000000000000276 (PMC10004749; doi:10.1097/PG9.0000000000000276)
Supplement: Supplementary file 1 [file pg9-4-e276-s001.pdf]

JPGN Reports

A Single Center Study of Long-Term Effectiveness of Vedolizumab in anti-TNF Refractory Pediatric Inflammatory Bowel Disease

--Manuscript Draft--

**Manuscript Number:**	JPGNREP-21-210R3
**Full Title:**	A Single Center Study of Long-Term Effectiveness of Vedolizumab in anti-TNF Refractory Pediatric Inflammatory Bowel Disease
**Article Type:**	Original Article
**Section/Category:**	Gastroenterology (North America)
**Keywords:**	vedolizumab, pediatrics, inflammatory bowel disease
**Corresponding Author:**	Halee Patel<br>Baylor College of Medicine<br>Houston, Texas UNITED STATES
**Corresponding Author Secondary Information:**	
**Corresponding Author's Institution:**	Baylor College of Medicine
**Corresponding Author's Secondary Institution:**	
**First Author:**	Halee Patel
**First Author Secondary Information:**	
**Order of Authors:**	Halee Patel
	Lina Karam
	Richard Kellermayer
**Order of Authors Secondary Information:**	
**Manuscript Region of Origin:**	UNITED STATES
**Abstract:**	Objectives: Vedolizumab is an anti-α4β7 integrin antibody that has been used successfully in the treatment of adult-onset inflammatory bowel diseases (IBDs: Crohn's disease [CD] and ulcerative colitis [UC]). Its off-label use in the pediatric IBD (PIBD) population is increasing, but knowledge on durability beyond 6 months of treatment is limited.<br>Methods: A real-life, single-center, retrospective study of PIBD patients treated with vedolizumab was performed. Data on demographics, prior and concomitant treatments, and disease activity were obtained at 14-weeks, 26-weeks, 1-year and 2-years of therapy. Primary outcome was corticosteroid and other biologic free remission (based on pediatric ulcerative colitis activity index [PUCAI]).<br>Results: Thirty-nine patients were studied. By 1-year, 65% of CD and 68% of UC patients continued on vedolizumab therapy. Corticosteroid and other biologic free remission was 29% in CD and 16% in UC. By 2-years, 36% of CD and 47% of UC patients continued therapy. Corticosteroid and other biologic free remission was 21% in CD and 40% in UC. By 2-years, 80% of CD and 100% of UC patients were on intensified treatment regimen compared to the manufacturer guidance. Nine patients (23%) required surgical intervention within 26 months of starting vedolizumab indicating the severity of IBD in this cohort.<br>Conclusions: Vedolizumab is a useful therapeutic modality in PIBD patients refractory to anti-TNF therapy, although with declining effectiveness by two years. Intensified treatment regimens are associated with long-term durability. Larger prospective trials in children are warranted.
**Additional Information:**	
**Question**	**Response**

Powered by Editorial Manager® and ProduXion Manager® from Aries Systems Corporation

Have any of the data and/or the subjects in the submitted manuscript been previously reported in another publication?	No
What is the word count of your manuscript? (word count applies only to main text; it does not include the abstract, what is new/known, references, or legends)	3141
How many figures and tables are included in this manuscript?	4

Powered by Editorial Manager® and ProduXion Manager® from Aries Systems Corporation

Texas Children's Hospital
6701 Fannin Street
MC CC1010.00
Houston, TX 77030-2299

Gastroenterology, Hepatology and Nutrition
Mission Statement
To strive for outstanding and compassionate patient car
through evidence-based and expert practice achieved throu
research and education

Baylor College of Medicine
www.bcm.edu
Department of Pediatrics
One Baylor Plaza
Houston, Texas 77030-3411

FACULTY

Richard Kellermayer
Associate Professor of Pediatrics
Baylor College of Medicine
Texas Children's Hospital

Benjamin L. Shneider, M.D.
Chief of Section
Anthony O. Anani, MD, MBA
Beth A. Carter, M.D.
Eric H. Chiou, M.D.
Andrew Chu, M.D.
Bruno P. Chumpitazi, M.D., MPH
Douglas S. Fishman, M.D., FASGE
Donna K. Garner, RN, MSN, PNP
Caroyl Gilbert, RN, MSN, PNP
G. S. Gopalakrishna, M.D.
Sanjiv Harpavat, M.D., Ph.D.
Paula M. Hertel, M.D.
Ryan W. Himes, M.D.
John M. Hollier, M.D.
Faith D. Ihekweazu, M.D.
Craig L. Jensen, M.D.
Lina B. Karam, M.D.
Richard Kellermayer, M.D., Ph.D.
Kristi L. King, M.P.H., R.D., L.D.
Seiji Kitagawa, M.D.
Daniel H. Leung, M.D.
Jennifer M. Maupin, MS, RN, CPNP
Carmen Mikhail, Ph.D.
Tamir Miloh, MD
Kathleen J. Motil, M.D., Ph.D.
Krupa Mysore, M.D.
Anthony P. Olivé, M.D.
Yen Pham, M.D.
Sarah M. Phillips, M.S., R.D.
Priya Raj, M.D., M.S.
Carol A. Redel, MNS, M.D.
Ann O. Scheimann, M.D.
Vernisha Y. Shepard, M.Ed., LPC
Robert J. Shulman, M.D.
Mary Elizabeth Tessier, MD
Sundararajah Thevananther, Ph.D.
Nicole Triggs,MSN,APRN,CPNP,CPN
Kristin L. Van Buren, M.D.
Bryan S. Vartabedian, M.D.
Seema Mehta Walsh, M.D.
Allyson E. Wyatt, M.D.

TELEPHONE
Office:

Office Fax:	832-825-3633
Clinic:	832-822-3131
Clinic Fax:	832-825-4131
Appointments:	832-822-2778

OUTREACH CLINICS

Sugar Land	281-494-7010
Cy-Fair	281-469-4688
Clearlake	281-282-1900
West Campus	832-227-1440
Woodlands	936-321-0808

September 13th, 2022

Re: A Single Center Study of Long-Term Effectiveness of Vedolizumab in anti-TNF Refractory Pediatric Inflammatory Bowel Disease

Dear Editors,

Thank you for the opportunity to revise and edit our manuscript entitled “A Single Center Study of Long-Term Effectiveness of Vedolizumab in anti-TNF Refractory Pediatric Inflammatory Bowel Disease” by Halee Patel, MD, Lina Karam, MD and Richard Kellermayer, MD. We appreciate the careful review of our manuscript and the constructive suggestions for improvement and modification. We do believe that the revised manuscript presents better quality results following the edits.

Following this letter are the reviewer and editor comments with our responses in italics. The revision has been developed in consultation with all coauthors, and each author has given approval to the final form of this revision.

We hope you find our revised manuscript suitable for publication and look forward to your positive decision.

Sincerely,

Richard Kellermayer M.D., Ph.D.
Associate Professor
Director, Pediatric Inflammatory Bowel Disease Program
Baylor College of Medicine, Texas Children's Hospital
6621 Fannin St., CC1010.00
Houston, TX 77030-2399
Voice: 713-798-0319; Fax: 713-798-0338

Reviewer Comments:

Reviewer 1: The revisions have resulted in an improved paper. Use of PUCAI in the patients with Crohn's disease remains a bit of a concern, but this is addressed as well as possible in both the methods and limitations and the authors have added a reference that had used a similar approach.

Thank you for the kind and positive feedback.

Editor Comments:

Thank you for your revision, now acceptable.
Please see the attachment with suggested edits - if you agree, please upload a revision with these changes for final acceptance!

Thank you for the reviewing the manuscript carefully. We have uploaded a revision with the suggested edits as requested.

A Single Center Study of Long-Term Effectiveness of Vedolizumab in anti-TNF Refractory Pediatric Inflammatory Bowel Disease

Halee Patel[1], Lina Karam[1], and Richard Kellermayer[1, 2*]

[1]Department of Pediatrics, Section of Gastroenterology, Hepatology and Nutrition, Baylor College of Medicine/Texas Children's Hospital, Houston, TX; [2]Children's Nutrition and Research Center, Houston, TX

Conflicts of Interest and Source of Funding: L.K. reports Primary Investigator status on Takeda funded clinical study. The study was supported in part by grant NIH T32 DK007664-29.

Acknowledgements: RK was supported in part by philanthropic funds from the Brock Wagner Family led Gutsy Kids Fund including the Frugoni family and other generous donors, and by the Klaasmeyer family funds for PSC research, as well as the ProKIIDS Network of the Crohn's and Colitis Foundation.

Contributions: H.P. collected and analyzed data, wrote manuscript draft; L.K. provided critical review of the manuscript and contributed to the final submission; R.K. performed conceptual design, data analysis, and manuscript writing

*Address for Correspondence: Halee Patel, MD, Section of Pediatric Gastroenterology, Hepatology & Nutrition, Baylor College of Medicine, 6701 Fannin St., MC1010.00, Houston, TX 77030-2399, Voice: 405-824-3900 Fax: 832-825-3633 Email: patelhalee@gmail.com

Total Word Count of Manuscript: 3141
Number of Figures: 2
Number of Tables: 2

Abstract

Objectives: Vedolizumab is an anti-α4β7 integrin antibody that has been used successfully in the treatment of adult-onset inflammatory bowel diseases (IBDs: Crohn's disease [CD] and ulcerative colitis [UC]). Its off-label use in the pediatric IBD (PIBD) population is increasing, but knowledge on durability beyond 6 months of treatment is limited.

Methods: A real-life, single-center, retrospective study of PIBD patients treated with vedolizumab was performed. Data on demographics, prior and concomitant treatments, and disease activity were obtained at 14-weeks, 26-weeks, 1-year and 2-years of therapy. Primary outcome was corticosteroid and other biologic free remission (based on pediatric ulcerative colitis activity index [PUCAI]).

Results: Thirty-nine patients were studied. By 1-year, 65% of CD and 68% of UC patients continued on vedolizumab therapy. Corticos~~S~~teroid and other biologic free remission was 29% in CD and 16% in UC. By 2-years, 36% of CD and 47% of UC patients continued therapy. Corticos~~S~~teroid and other biologic free remission was 21% in CD and 40% in UC. By 2-years, 80% of CD and 100% of UC patients were on intensified treatment regimen compared to the manufacturer guidance. Nine patients (23%) required surgical intervention within 26 months of starting vedolizumab indicating the severity of IBD in this cohort.

Conclusions: Vedolizumab ~~was~~ is a useful therapeutic modality in PIBD patients refractory to anti-TNF therapy, although with declining effectiveness by two years. Intensified treatment regimens ~~were~~ are associated with long-term durability. Larger prospective trials in children are warranted.

Key Words: vedolizumab**,** pediatrics, inflammatory bowel disease

What is Known:

- Several pediatric studies have shown that vedolizumab may be safe and effective in anti-TNF refractory cases of inflammatory bowel disease (IBD).
- The effectiveness of vedolizumab has been studied in the adult population, but there is limited data on its long-term use and durability in children.

What is New:

- Long term efficacy of vedolizumab declines~~d~~ over two years in pediatric IBD (PIBD) patients.
- Intensified dosing of vedolizumab compared to the standard adult regimen ~~was~~ is progressively ~~used~~ needed to maintain~~-~~ therapeutic efficacy~~therapy~~.
- Our findings further support the ~~notion~~ concept that biologic pharmacokinetics may differ between adult and pediatric IBD patients.

Introduction

A continuing rise of pediatric inflammatory bowel disease (PIBD) incidence has been observed in several recent studies.[1-3] PIBD is frequently more aggressive than the adult onset and even the highly efficient anti-tumor necrosis factor-alpha (anti-TNF) biologic agents can fail primarily or over time (i.e. secondary failure).[4-6] Therefore, novel and optimized modes of treatment are critically needed in these patients, especially in those with anti-TNF therapy failure.

Vedolizumab is an anti-α4β7 integrin antibody with gut-selective anti-inflammatory activity that has been used successfully in the treatment of adult onset IBDs (Crohn's disease [CD] and ulcerative colitis [UC]).[7] Vedolizumab acts on the α4β7 integrin receptor on lymphocytes, blocking their interaction with MadCAM-1 on the intestinal endothelium, and thereby inhibiting lymphocyte migration to the intestinal mucosa. As this interaction is gut-selective, the risks of systemic immunosuppression that were seen with the drug's predecessor (natalizumab) on the central nervous system are significantly decreased.[8]

The clinical trials of GEMINI 1, 2, and 3 demonstrated the durability of vedolizumab in adult patients, notably with better results in UC compared to CD.[8-10] More recently, the Cross Pennine study in adults demonstrated the long-term effectiveness and appropriate safety profile of vedolizumab; ~~with~~ 78.5% of CD patients and 91.2% of UC patients ~~who had~~showed clinical response or remission at 14 weeks, ~~whereas~~ and 63.9% of CD and ~~91.2~~82.5% of UC patients continued to show response or remission at 52 weeks.[11]

The off-label use of vedolizumab and data on its efficacy in PIBD are increasing. Singh *et al.* in 2016 reported that at week 14, 42% of CD patients, and 76% of UC patients were in clinical remission (n= 52).[8] This study also found improved remission rates for anti-TNF naïve

patients ~~compared~~ in contrast to patients with previous exposure of anti-TNF agents (100% n=4 versus 45% n=28, p= 0.04). Safety of vedolizumab was also indicated in this work. Several other pediatric studies ~~including Conrad *et al.*,[12] Ledder *et al.*,[13] and Schneider *et al.*[14] have~~ demonstrated that vedolizumab is safe and effective for use in PIBD up to week 22[12-13] and week 38[14] of treatment. ~~More recently,~~ Hajjat *et al.* ~~performed~~ published a multicenter retrospective study in 2021 in which 43% of pediatric patients were observed to achieve corticosteroid free remission on vedolizumab at 1 year.[15] Additionally, data from the phase 2 HUBBLE study ~~were recently published by Hyams *et al.* which noted~~revealed that vedolizumab serum concentrations increased in a dose-proportional manner, ~~but there was~~although no clear dose-response relationship was observed.[16] This study was limited in its sample size, but was the first to report ~~on~~ pharmacokinetic data for vedolizumab use in children~~ to date~~. ~~Taking these studies into consideration, there is still limited~~ Thus, data are limited regarding the effectiveness of vedolizumab beyond 6 months of treatment. We aimed to examine the long-term ~~effectiveness~~ efficacy of vedolizumab therapy in our pediatric population at a tertiary PIBD center.

Materials and Methods:

Pediatric patients who were initiated on vedolizumab at Texas Children’s Hospital in Houston, TX between September 2015 and September 2018 and completed the induction phase of treatment (through week 14) were included in this study. The decision to initiate vedolizumab was at the discretion of the treating physician. Pertinent data were collected through the end of the study period in September 2020 if available. The study was approved by the Institutional Review Board of Texas Children’s Hospital, Baylor College of Medicine (H-43380).

Age at diagnosis, age at vedolizumab initiation, previous or concomitant corticosteroid, biological or immunomodulatory therapy, disease activity, and surgical history were collected. Disease activity was defined by the Pediatric Ulcerative Colitis Activity Index (PUCAI) and retrospectively calculated by chart review for all patients, including patients with CD. Due to the retrospective nature of this study, several data required for calculating the Pediatric Crohn's Disease Activity Index (PCDAI) and even the abbreviated PCDAI wereas unavailable. Therefore, we decided to calculate PUCAI scores for all patients, including those with CD, as the primary burden of their disease was colonic/ileocolonic,. This approach to optimize disease activity assessment in retrospective studies on CD by PUCAI scoringas has been done in prior studies as well.[17] Data on laboratory biomarkers or endoscopic evaluations were not routinely available at the required timepoints and therefore were not evaluated in this study. Data on disease activity specifically were focused at 14-weeks, 26-weeks, 1-year and 2-years of therapy. Dosing regimens including the frequency of infusions were notedrecorded. Vedolizumab drug levels and timing of therapeutic drug monitoring (TDM) were noted documented if available. Data on adverse events were also collected.

The primary outcome of the study was defined as corticosteroid and other biologic agent free remission at 26-weeks and 1-year. Clinical remission was defined as a PUCAI score of less than 10. Mild disease activity was defined as a PUCAI of 10-34 and moderate/severe disease activity was defined as a PUCAI > 35.[18] Secondary outcomes included discontinuation of therapy, corticosteroid and other biologic agent free remission at 14-weeks and 2-years of treatment, need for surgical intervention, and the time from initiation of vedolizumab to surgical intervention.

Data were reported in percentage of patients achieving remission and were compared across independent groups by using Fischer's exact test. Statistical significance level was set at $p<0.05$.

Results

Patient Characteristics

A total of 39 patients completed the initial induction treatment of vedolizumab for CD (49%) or UC (~~49% for CD and~~ 51%~~ for UC~~)~~ at Texas Children's Hospital between September 2015 and September 2018~~. Data on baseline characteristics are reported in Table 1. The predominant CD phenotype was L3/B1 (ileocolonic [63%], non-stricturing, non-penetrating) according to Paris classification.[19] The predominant UC phenotype was pancolitis (E4, 95%) with 100% having an episode of ever having severe disease (PUCAI score > 65). The mean age at initiation of vedolizumab was 14.5 years with an age range of 5-19. Thirty-eight of the 39 patients (97%) were refractory to previous anti-TNF therapy (defined as having an inadequate response to the agent as primary non-response, or secondary loss of response, or adverse reaction). Only 1/39 (3%) patient with CD (L2) was anti-TNF naïve, who was initiated on vedolizumab after strict specific carbohydrate diet and oral vancomycin therapy failed. Overall, 16 (41%) of the patients had been treated with a second anti-TNF agent (adalimumab) prior to vedolizumab.

Details of Vedolizumab Induction

O~~Out o~~f the 39 patients who completed the initial three dose induction of vedolizumab, 20 (51%) received the "standard" induction regimen (i.e. according to manufacturer

recommendation) with vedolizumab infusions administered at 0, 2, 6 and 14 weeks. The remaining 19 patients underwent a modified induction at their primary gastroenterologist's discretion in response to either persistent or worsening symptoms (reactive change in infusion schedules), or prospectively, based on subjective clinical experience. Fourteen of these patients had interval change compared to the standard after the third (week 6) infusion and the remaining five patients had intensification prior to week 6 of therapy.

Combination treatment regimens during induction varied among ~~st~~ the patients ~~as well~~; 7 patients with CD (37%) and 9 patients with UC (45%) were given corticosteroids alone for induction but 16% of patients with CD and 25% of patients with UC were concomitantly on another agent such as a biologic or immunomodulator in addition to the corticosteroids. Dual biologic therapy was used in 8 patients with CD (42%) which included 6 patients on adalimumab, 1 patient on infliximab, and 1 patient on ustekinumab. Dual biologic therapy was used in 5 patients with UC (25%) during induction ~~as well~~, which included 3 patients on adalimumab and 2 patients on infliximab. These patients remained on their prior biologic agent as bridge therapy while undergoing induction with vedolizumab ~~per~~ at the discretion of their primary gastroenterologist. Data for induction regimens are summarized in Supplementary Table 1.

Thirty-three (85%) patients received the standard, adult dose of vedolizumab (300 mg) and the remaining 6 patients received ~ 6 mg/kg dose (ranging from 100 mg to 200 mg per dose). The youngest patient to receive the 300 mg dose was 9 years old at the start of vedolizumab.

Clinical Remission on Vedolizumab

Data were available on 36/39 patients (92%) at 26 weeks and 1 year after initiation of vedolizumab (Supplementary Figure 1). Two-year data were available for 29/39 patients (74%). At week 14, 26% (5/19) of CD and 60% (12/20) of UC patients achieved clinical remission and 11% (2/19) of CD and 45% (9/20) of UC patients achieved both corticosteroid and other biologic free remission (Figures 1 and 2). At week 26, 24% (4/17) of CD and 32% (6/19) of UC patients achieved clinical remission and 18% (3/17) of CD and 32% (6/19) of UC patients achieved corticosteroid and other biologic free remission, respectively. At 1-year, 29% (5/17) of CD and 16% (3/19) of UC patients achieved corticosteroid and other biologic free remission. At 2-years, 21% (3/14) of CD and 40% (6/15) of UC patients had achieved clinical remission without requiring any corticosteroid or other biologic agents. At 2-years, only 4 (2 CD, 2 UC) patients were receiving vedolizumab monotherapy (including no immunomodulator or salicylate therapy).

Details on Dual Biologic Use

Patients who received dual biologic therapy (vedolizumab plus another biologic agent) at 14-weeks, 26-weeks, or 1-year did not demonstrate a significant difference in clinical remission or corticosteroid free remission rates (p>0.1) in comparison tocompared with patients on vedolizumab monotherapy. However, IBD patients that were not on dual biologic agents at 2-years were more likely to be in corticosteroid free clinical remission when compared withto IBD patients requiring vedolizumab plus another biologic agent (p=0.004). Combination regimens are further described in Table 2.

Out ofOf the 8 patients with CD who continued to receive another biologic agent during the induction of vedolizumab, 13% (1/8) achieved clinical remission at week 14 but 75% (6/8)

continued to ~~note~~ have mild disease activity. The one patient who achieved clinical remission at week 14 was on ustekinumab concomitantly during induction and maintenance but ultimately was taken off ~~of~~ vedolizumab before reaching the 1-year timepoint. Furthermore, ~~out~~ of the mild disease activity group, 1 patient ultimately achieved clinical remission by 26-weeks and remained on vedolizumab monotherapy by 1-year and 2-years post vedolizumab initiation.

~~Out of~~Of the 5 patients with UC who continued to receive an anti-TNF agent during the induction of vedolizumab, 60% (3/5) achieved corticosteroid free clinical remission at week 14. Two of the patients were on adalimumab during induction and remained on adalimumab through 26-weeks and 1-year but thereafter developed mild disease activity despite the dual biologic therapy. Furthermore, the remaining patient who had achieved corticosteroid free clinical remission at week 14 was on infliximab during induction but was taken off of infliximab at week 14. This patient continued to remain in corticosteroid free clinical remission at the 2-year timepoint on vedolizumab monotherapy.

A second biologic agent was not added for any patients with UC who were initiated on vedolizumab monotherapy. However, 3 patients with CD who had undergone standard induction with vedolizumab alone required the addition of a second biologic agent at or after 1 year of the monotherapy. One of these patients received infliximab but ultimately stopped vedolizumab therapy. The other 2 patients received ustekinumab in addition to vedolizumab and ultimately only one of the two patients was able to remain on dual ustekinumab/vedolizumab therapy at 2 years but with continued mild disease activity.

~~There was no significant difference (p>0.1) in~~ Corticosteroid free remission rates in patients with colonic only disease (including UC and colonic only CD) ~~in comparison to~~was no different compared with patients with SB involvement (ileocolonic CD) at both 26-week (colonic

only 50% versus ileocolonic 50%) and 1-year (colonic only 58% versus ileocolonic 50%) timepoints. ~~There was no difference in~~ C~~c~~linical remission or corticosteroid plus other biologic agent free remission rates also did not differ~~either~~.

~~There was no~~No significant (p>0.1) difference ~~in respect~~was found relating to gender in any of the outcomes examined.

Durability of Vedolizumab

At week 26, 76% (13/17) of patients with CD and 74% (14/19) with UC remained on therapy (Figures 1 and 2). Of the patients with 52-week outcomes available, 65% (11/17) with CD and 68% (13/19) with UC remained on vedolizumab. By 2-years, 9 patients with CD and 8 patients with UC had discontinued vedolizumab therapy due to severity of disease. No significant (p>0.05) difference between CD or UC was ~~noted~~ found in any of these outcomes.

Among the patients remaining on vedolizumab at 1-year, 64% of CD patients (4-week interval n=3, 6-week interval n= 4) and 85% of UC patients (4-week interval n= 6, 6-week interval n= 5) were on an intensified regimen compared ~~to~~ with the adult conventional dosing of every 8-week infusions (Supplementary Figure 2). This ratio of intensified treatment increased to 80% of CD (4-week n=3, 6-week n=1) and 100% of UC patients (4-week n=5, 6-week n=2) by 2-years. No significant (p>0.05) difference between standard versus intensified dosing regimens was ~~noted~~ observed in any of these outcomes.

Surgical Outcomes

Seven patients with UC (35%) and 2 patients with CD (10%) required surgical intervention (partial/total colectomy or diverting ileostomy) following initiation of vedolizumab.

The time from initiation of vedolizumab to surgical intervention varied from 3 months to 26 months (median time of 14 months). Among~~st~~ all~~ the~~ patients who required surgical intervention, 3 patients were in clinical remission by week 14, ~~out ~~of which 2 patients also met criteria for corticosteroid free remission~~ as well~~. However, by 1-year, only 2 patients remained in corticosteroid free remission. Four patients required surgical intervention in less than 1 year from the start of vedolizumab. All four of these patients were noted to have mild to moderate disease activity at week 14 and 75% (3/4) of the patients subsequently had discontinued vedolizumab before reaching week 26. The remaining~~ one~~ patient had undergone a diverting ileostomy at 12-weeks following initiation of vedolizumab but was able to enter clinical remission by 26-weeks and continued to remain in corticosteroid free remission at the 1-year timepoint.

Therapeutic Drug Monitoring

Twenty 20 patients (51%) (9 CD and 11 UC) had therapeutic drug monitoring (TDM: vedolizumab levels and antibodies). ~~, of which 9 patients had CD and 11 had UC.~~ TDM timing was not standardized and the decision to obtain vedolizumab levels and antibodies was directed by the individual physicians. None of these patients developed antibodies to vedolizumab. Meaningful analyses could not be performed on vedolizumab TDM in this cohort due to the inconsistency in the timing.~~ of that.~~

Patient safety

No serious adverse reactions to vedolizumab during the observation period were reported. One mild, possible drug related event was reported in a patient who developed nausea and

vomiting immediately following the 4th dose and was thereby discontinued from further therapy of vedolizumab.

Discussion

~~This is~~We report the largest real-life PIBD cohort treated with vedolizumab with 1-year and 2-year outcomes to date. Response to vedolizumab~~,~~ or the effectiveness of this biologic decreased over time. Other pediatric studies generally report~~ed~~ similar findings, but have not examined 2-year outcomes.[8,12-15] In the 2017 study by Ledder, *et al.*, at week 14, 25% of CD patients (n=16) and 47% of UC patients (n=34) were in corticosteroid free clinical remission. At week 22, 36% of CD (n =14) and 46% of UC patients (n=26) were in clinical remission. This study also reported 1-year data with 25% (1/4) of CD patients and 60% (6/10) of UC patients in clinical remission and ultimately noted that vedolizumab was effective, especially in UC, in inducing and maintaining remission during long-term use.[13] ~~In the meantime, the~~ UC specific ~~effectiveness~~ efficacy of vedolizumab did not reach statistical significance compared with~~to~~ CD (p=0.56). Our study supports this latter result~~,~~ since no ~~significant~~ difference in outcomes between patients with CD and UC patients was observed. This finding underscores the importance of independent, larger cohort examinations of biologic effectiveness in PIBD. Our results ~~are~~ also ~~similar to~~reflect adult trial outcomes, which report~~ed~~ 32% of CD patients and 39% of UC patients in corticosteroid free clinical remission at 1-year, although only 16% of our UC patients were in clinical remission at the same time point.[7] Our cohort also included several patients who received dual biologic therapy from the initiation or during the maintenance phases. ~~Interestingly~~Noteworthy, patients who were on dual biologic agents at 2-years were less likely to be in clinical remission ~~in comparison~~compared with ~~to~~ patients on single biologic therapy

(p=0.004). This alludes to the disease severity of our cohort. Similar to other studies,As reported by others, we did not find any serious adverse reaction to vedolizumab.

The majorityMost of our patients who were maintained on vedolizumab past 1-year and 2-year timepoints required interval intensification. All patients with UC at 2-years were on an intensified regimen. These findings suggest that intensified regimens may support the long-term maintenance of vedolizumab therapy. Intensified therapy of biologic agents in PIBD is becoming increasingly more common when compared withto standard adult practices.[18] In a recent study by *Jongsma et al.*, recently reported that younger pediatric patients on infliximab were significantly more likely to be on intensified therapy to maintain clinical remission at 1-year.[20] Similar results have been observed for other biologics such as adalimumab[21] and ustekinumab[22] in the pediatric population. Our study also favors the use of intensified therapy for maintenance of vedolizumab in PIBD.

With increasing availability and ease of testing, TDM for other biologic agents such as adalimumab and infliximab in pediatric patients is becoming more common as a tool forto optimize optimizing treatment and potentially clinical outcomes.[23] With regards to TDM of vedolizumab, it has been indicated as a useful tool in adult patients.[24] However, in the pediatric population, data on TDM for vedolizumab are limited to one study recently published by Aardoom, *et al.* who concluded that patients with CD may benefit from routine TDM and intensified dosing regimens.[25] The limited data on vedolizumab TDM in the pediatric population is likely due to the lack of standardized level testing in clinical practice. Surely, pharmaceutically supported Pprospective studies on pediatric pharmacokinetics for vedolizumab such as reported the recently completed study by Hyams *et al.* will further our understanding on optimized use of vedolizumab and TDM in PIBD.[16]

Although the regional/single center nature of this work may be considered a limitation, Shiau *et al.*[26] have suggested that consistency of medical care in single centers may improve the accuracy of clinical studies in IBD. A recent study from the largest prospective cohort on PIBD patients has underscored the significant variation in clinical care (including diagnosis and treatment) between among the North American medical centers involved.[27] This work supports our premise on single center studies potentially providing higher accuracy when examining questions on management in PIBD even with smaller sample sizes than in multi-center cohorts. Regardless, our single center study also calls for standardized approaches in inwith respect to TDM (by highlighting the lack thereof in real-life practice at a single center).

This study is limited by its retrospective nature and therefore in the ability to control for treatment regimens as well asand for testing and follow up. PUCAI scores were used in all patients, including those with CD, due to limited data available and inability to calculate CD specific scores for disease activity (PCDAI). Although the largest single center cohort of its kind, this work is limited by its cohort size and the lack of standardized measures for TDM use as highlighted above. Furthermore, our study included CD patients with only ileocolonic or colonic predominant disease. Therefore, our data cannot be extrapolated for CD patients with small bowel only or upper GI gastrointestinal disease.

Our observations indicate that vedolizumab iswas safe, but its overall efficacy declinesd with time in anti-TNF exposed CD and UC patients. Our findings also favor the need for intensified treatment regimens of vedolizumab in PIBD to promote long term maintenance of therapy. These findings emphasize the need for prospective optimization of treatment with vedolizumab and the ongoing requirement for novel preventative and therapeutic measures to combat this highly morbid disease group.

References:

1. Nasiri S, Kuenzig ME, and Benchimol, E. Long-term outcomes of pediatric inflammatory bowel disease. *Semin Pediatr Surg* 2017;26:398-404.
2. Ng S, Shi H, Hamidi N, *et al*. Worldwide incidence and prevalence of inflammatory bowel disease in the 21st century: a systematic review of population-based studies. *Lancet* 2018;390:2769-2778.
3. Sykora J, Pomahacova R, Kreslova M, *et al*. Current global trends in the incidence of pediatric-onset inflammatory bowel disease. *World J Gastroenterol* 2018;24:2741-2763.
4. Sonavane AD, Sonawane P, and Amarapurkar DN. Inflammatory bowel disease across the age continuum: similarity and disparity. *Indian J Pediatr* 2018;85:989-994.

5. Assa A, Bronsky J, Kolho KL, *et al*. Anti-TNF[alpha] treatment after surgical resection for Crohn's Disease is effective despite previous pharmacodynamics failure. *Inflamm Bowel Dis* 2017;23:791-797.
6. Ihekweazu F, Fofanova T, Palacios R, *et al.* Progression to colectomy in the era of biologics: A single center experience with pediatric ulcerative colitis. *J Pediatr Surg* 2020;55:1815-1823.
7. Engel T, Ungar B, Yung DE, *et al*. Vedolizumab in IBD – Lessons from real-world experience; a systematic review and pooled analysis. *J Crohn's Colitis* 2018;12:245-257.
8. Singh N, Rabizadeh S, Jossen J, *et al*. Multi-center experience of vedolizumab effectiveness in pediatric inflammatory bowel disease. *Inflamm Bowel Dis* 2016;22:2121-2126.
9. Feagan BG, Rutgeerts P, Sands BE, *et al*. Vedolizumab as induction and maintenance therapy for ulcerative colitis. *N Engl J Med* 2013;369:699-710.
10. Sandborn WJ, Feagan BG, Rutgeerts P, *et al*. Vedolizumab as induction and maintenance therapy for Crohn's disease. *N Engl J Med* 2013;369:711-721.
11. Lenti MV, Levison S, Eliadou E, *et al*. A real-world, long-term experience on effectiveness and safety of vedolizumab in adult patients with inflammatory bowel disease: The Cross Pennine study. *Dig Liver Dis* 2018;50:1299-1304.
12. Conrad MA, Stein RE, Maxwell EC, *et al*. Vedolizumab therapy in severe pediatric inflammatory bowel disease. *Inflamm Bowel Dis* 2016;22:2425-2431.
13. Ledder O, Assa A, Levine A, *et al*. Vedolizumab in paediatric inflammatory bowel disease: a retrospective multi-centre experience from the paediatric IBD Porto group of ESPGHAN. *J Crohn's Colitis* 2017;11:1230-1237.

14. Schneider A, Weghuber D, Hetzer B, *et al*. Vedolizumab use after failure of TNF-∝ antagonists in children and adolescents with inflammatory bowel disease. *BMC Gastroenterol* 2018;18:140.

15. Hajjat TM, Mosha M, Whaley KG, *et al.* Vedolizumab experience in children and adolescents with inflammatory bowel disease: a multicenter observational study. *Crohns & Colitis 360.* 2021;3:1-8.

16. Hyams JS, Turner D, Cohen SA, *et al.* Pharmacokinetics, safety, and efficacy of intravenous vedolizumab in paediatric patients with ulcerative colitis or Crohn's disease: results from the phase 2 HUBBLE study. *J Crohns Colitis.* 2022. doi: 10.1093/ecco-jcc/jjac036. [Epub ahead of print].

17. Breton J, Kastl A, Hoffman N, *et al.* Efficacy of combination antibiotic therapy for refractory pediatric inflammatory bowel disease. *Inflamm Bowel Dis.* 2019;25:1586-1593.

18. Turner D, Otley AR, Mack D, *et al.* Development, validation, and evaluation of a pediatric ulcerative colitis activity index: a prospective multicenter study. *Gastroenterology.* 2007;133:423–432.

19. Assa A, Rinawi F, and Shamir R. The long-term predictive properties of the Paris classification in pediatric inflammatory bowel disease patients. *J Crohn's Colitis* 2018;12:39-47.

20. Jongsma M, Winter DA, Huynh HQ, *et al.* Infliximab in young paediatric IBD patients: it is all about the dosing. *Eur J Pediatr* 2020;179:1935-1944.

21. Croft NM, Faubion Jr WA, Kugathasan S, *et al.* Efficacy and safety of adalimumab in paediatric patients with moderate-to-severe ulcerative colitis (ENVISION I): a randomised, controlled, phase 3 study. *Lancet Gastroenterol Hepatol* 2021;6:616-627.
22. Do P, Andersen J, Patel A, *et al.* Augmented ustekinumab dosing is needed to achieve clinical response in patients with anti-TNF refractory pediatric Crohn's disease: a retrospective chart review. *F1000Res* 2020;9:316.
23. Carman N, Mack DR, and Benchimol EI. Therapeutic drug monitoring in pediatric inflammatory bowel disease. *Curr Gastroenterol Rep* 2018;20:18.
24. Ward MG, Sparrow MP and Roblin X. Therapeutic drug monitoring of vedolizumab in inflammatory bowel disease: current data and future directions. *Therap Adv Gastroenterol* 2018;11:1-10.
25. Aardoom A, Jongsma M, de Vries A, *et al.* Vedolizumab trough levels in children with anti-TNF refractory inflammatory bowel disease. *J Pediatr Gastroenterol Nutr* 2020;71:501-507.
26. Shiau H, Ihekweazu FD, Amin M, *et al*. The unique inflammatory bowel disease phenotype of pediatric primary sclerosing cholangitis: a single center study. *J Pediatr Gastroenterol Nutr* 2017;65:404-409.
27. Krishnakumar C, Ballengee CR, Liu C, *et al*. Variation in care in the management of children with Crohn's disease: data from a multicenter inception cohort study. *Inflamm Bowel Dis* 2019;25:1208-1217.

Figure Legend

Figure 1: Disease activity and clinical outcomes in patients with Crohn's disease on vedolizumab treatment at 14-weeks, 26-weeks, 1-year and 2-years.

Figure 2: Disease activity and clinical outcomes in patients with ulcerative colitis on vedolizumab treatment at 14-weeks, 26-weeks, 1-year and 2-years.

Abstract

Objectives: Vedolizumab is an anti-α4β7 integrin antibody that has been used successfully in the treatment of adult-onset inflammatory bowel diseases (IBDs: Crohn's disease [CD] and ulcerative colitis [UC]). Its off-label use in the pediatric IBD (PIBD) population is increasing, but knowledge on durability beyond 6 months of treatment is limited.

Methods: A real-life, single-center, retrospective study of PIBD patients treated with vedolizumab was performed. Data on demographics, prior and concomitant treatments, and disease activity were obtained at 14-weeks, 26-weeks, 1-year and 2-years of therapy. Primary outcome was corticosteroid and other biologic free remission (based on pediatric ulcerative colitis activity index [PUCAI]).

Results: Thirty-nine patients were studied. By 1-year, 65% of CD and 68% of UC patients continued on vedolizumab therapy. Corticosteroid and other biologic free remission was 29% in CD and 16% in UC. By 2-years, 36% of CD and 47% of UC patients continued therapy. Corticosteroid and other biologic free remission was 21% in CD and 40% in UC. By 2-years, 80% of CD and 100% of UC patients were on intensified treatment regimen compared to the manufacturer guidance. Nine patients (23%) required surgical intervention within 26 months of starting vedolizumab indicating the severity of IBD in this cohort.

Conclusions: Vedolizumab is a useful therapeutic modality in PIBD patients refractory to anti-TNF therapy, although with declining effectiveness by two years. Intensified treatment regimens are associated with long-term durability. Larger prospective trials in children are warranted.

Key Words: vedolizumab**,** pediatrics, inflammatory bowel disease

What is Known:

- Several pediatric studies have shown that vedolizumab may be safe and effective in anti-TNF refractory cases of inflammatory bowel disease (IBD).
- The effectiveness of vedolizumab has been studied in the adult population, but there is limited data on its long-term use and durability in children.

What is New:

- Long term efficacy of vedolizumab declines over two years in pediatric IBD (PIBD) patients.
- Intensified dosing of vedolizumab compared to the standard adult regimen is progressively needed to maintain therapeutic efficacy.
- Our findings further support the concept that biologic pharmacokinetics may differ between adult and pediatric IBD patients.

Introduction

A continuing rise of pediatric inflammatory bowel disease (PIBD) incidence has been observed in several recent studies.[1-3] PIBD is frequently more aggressive than the adult onset and even the highly efficient anti-tumor necrosis factor-alpha (anti-TNF) biologic agents can fail primarily or over time (i.e. secondary failure).[4-6] Therefore, novel and optimized modes of treatment are critically needed in these patients, especially in those with anti-TNF therapy failure.

Vedolizumab is an anti-α4β7 integrin antibody with gut-selective anti-inflammatory activity that has been used successfully in the treatment of adult onset IBDs (Crohn's disease [CD] and ulcerative colitis [UC]).[7] Vedolizumab acts on the α4β7 integrin receptor on lymphocytes, blocking their interaction with MadCAM-1 on the intestinal endothelium, and thereby inhibiting lymphocyte migration to the intestinal mucosa. As this interaction is gut-selective, the risks of systemic immunosuppression that were seen with the drug's predecessor (natalizumab) on the central nervous system are significantly decreased.[8]

The clinical trials of GEMINI 1, 2, and 3 demonstrated the durability of vedolizumab in adult patients, notably with better results in UC compared to CD.[8-10] More recently, the Cross Pennine study in adults demonstrated the long-term effectiveness and appropriate safety profile of vedolizumab; 78.5% of CD patients and 91.2% of UC patients showed clinical response or remission at 14 weeks, and 63.9% of CD and 82.5% of UC patients continued to show response or remission at 52 weeks.[11]

The off-label use of vedolizumab and data on its efficacy in PIBD are increasing. Singh *et al.* in 2016 reported that at week 14, 42% of CD patients, and 76% of UC patients were in clinical remission (n= 52).[8] This study also found improved remission rates for anti-TNF naïve

patients in contrast to patients with previous exposure of anti-TNF agents (100% n=4 versus 45% n=28, p= 0.04). Safety of vedolizumab was also indicated in this work. Several other pediatric studies demonstrated that vedolizumab is safe and effective for use in PIBD up to week 22[12-13] and week 38[14] of treatment. Hajjat *et al.* published a multicenter retrospective study in 2021 in which 43% of pediatric patients were observed to achieve corticosteroid free remission on vedolizumab at 1 year.[15] Additionally, data from the phase 2 HUBBLE study revealed that vedolizumab serum concentrations increased in a dose-proportional manner, although no clear dose-response relationship was observed.[16] This study was limited in its sample size, but was the first to report pharmacokinetic data for vedolizumab use in children. Thus, data are limited regarding the effectiveness of vedolizumab beyond 6 months of treatment. We aimed to examine the long-term efficacy of vedolizumab therapy in our pediatric population at a tertiary PIBD center.

Materials and Methods:

Pediatric patients who were initiated on vedolizumab at Texas Children's Hospital in Houston, TX between September 2015 and September 2018 and completed the induction phase of treatment (through week 14) were included in this study. The decision to initiate vedolizumab was at the discretion of the treating physician. Pertinent data were collected through the end of the study period in September 2020 if available. The study was approved by the Institutional Review Board of Texas Children's Hospital, Baylor College of Medicine (H-43380).

Age at diagnosis, age at vedolizumab initiation, previous or concomitant corticosteroid, biological or immunomodulatory therapy, disease activity, and surgical history were collected. Disease activity was defined by the Pediatric Ulcerative Colitis Activity Index (PUCAI) and

retrospectively calculated by chart review for all patients, including patients with CD. Due to the retrospective nature of this study, several data required for calculating the Pediatric Crohn's Disease Activity Index (PCDAI) and even the abbreviated PCDAI were unavailable. Therefore, we decided to calculate PUCAI scores for all patients, including those with CD, as the primary burden of their disease was colonic/ileocolonic, as has been done in prior studies.[17] Data on laboratory biomarkers or endoscopic evaluations were not routinely available at the required timepoints and therefore were not evaluated in this study. Data on disease activity specifically were focused at 14-weeks, 26-weeks, 1-year and 2-years of therapy. Dosing regimens including the frequency of infusions were recorded. Vedolizumab drug levels and timing of therapeutic drug monitoring (TDM) were documented if available. Data on adverse events were also collected.

The primary outcome of the study was defined as corticosteroid and other biologic agent free remission at 26-weeks and 1-year. Clinical remission was defined as a PUCAI score of less than 10. Mild disease activity was defined as a PUCAI of 10-34 and moderate/severe disease activity was defined as a PUCAI > 35.[18] Secondary outcomes included discontinuation of therapy, corticosteroid and other biologic agent free remission at 14-weeks and 2-years of treatment, need for surgical intervention, and the time from initiation of vedolizumab to surgical intervention.

Data were reported in percentage of patients achieving remission and were compared across independent groups by using Fischer's exact test. Statistical significance level was set at $p<0.05$.

Results

Patient Characteristics

A total of 39 patients completed the initial induction treatment of vedolizumab for CD (49%) or UC (51%). Data on baseline characteristics are reported in Table 1. The predominant CD phenotype was L3/B1 (ileocolonic [63%], non-stricturing, non-penetrating) according to Paris classification.[19] The predominant UC phenotype was pancolitis (E4, 95%) with 100% having an episode of ever having severe disease (PUCAI score > 65). The mean age at initiation of vedolizumab was 14.5 years with an age range of 5-19. Thirty-eight of the 39 patients (97%) were refractory to previous anti-TNF therapy (defined as having an inadequate response to the agent as primary non-response, or secondary loss of response, or adverse reaction). Only 1/39 (3%) patient with CD (L2) was anti-TNF naïve, who was initiated on vedolizumab after strict specific carbohydrate diet and oral vancomycin therapy failed. Overall, 16 (41%) of the patients had been treated with a second anti-TNF agent (adalimumab) prior to vedolizumab.

Details of Vedolizumab Induction

Of the 39 patients who completed the initial three dose induction of vedolizumab, 20 (51%) received the "standard" induction regimen (i.e. according to manufacturer recommendation) with vedolizumab infusions administered at 0, 2, 6 and 14 weeks. The remaining 19 patients underwent a modified induction at their primary gastroenterologist's discretion in response to either persistent or worsening symptoms (reactive change in infusion schedules), or prospectively, based on subjective clinical experience. Fourteen of these patients had interval change compared to the standard after the third (week 6) infusion and the remaining five patients had intensification prior to week 6 of therapy.

Combination treatment regimens during induction varied among the patients; 7 patients with CD (37%) and 9 patients with UC (45%) were given corticosteroids alone for induction but 16% of patients with CD and 25% of patients with UC were concomitantly on another agent such as a biologic or immunomodulator in addition to the corticosteroids. Dual biologic therapy was used in 8 patients with CD (42%) which included 6 patients on adalimumab, 1 patient on infliximab, and 1 patient on ustekinumab. Dual biologic therapy was used in 5 patients with UC (25%) during induction, which included 3 patients on adalimumab and 2 patients on infliximab. These patients remained on their prior biologic agent as bridge therapy while undergoing induction with vedolizumab at the discretion of their primary gastroenterologist. Data for induction regimens are summarized in Supplementary Table 1.

Thirty-three (85%) patients received the standard, adult dose of vedolizumab (300 mg) and the remaining 6 patients received ~ 6 mg/kg dose (ranging from 100 mg to 200 mg per dose). The youngest patient to receive the 300 mg dose was 9 years old at the start of vedolizumab.

Clinical Remission on Vedolizumab

Data were available on 36/39 patients (92%) at 26 weeks and 1 year after initiation of vedolizumab (Supplementary Figure 1). Two-year data were available for 29/39 patients (74%). At week 14, 26% (5/19) of CD and 60% (12/20) of UC patients achieved clinical remission and 11% (2/19) of CD and 45% (9/20) of UC patients achieved both corticosteroid and other biologic free remission (Figures 1 and 2). At week 26, 24% (4/17) of CD and 32% (6/19) of UC patients achieved clinical remission and 18% (3/17) of CD and 32% (6/19) of UC patients achieved corticosteroid and other biologic free remission, respectively. At 1-year, 29% (5/17) of CD and

16% (3/19) of UC patients achieved corticosteroid and other biologic free remission. At 2-years, 21% (3/14) of CD and 40% (6/15) of UC patients had achieved clinical remission without requiring any corticosteroid or other biologic agents. At 2-years, only 4 (2 CD, 2 UC) patients were receiving vedolizumab monotherapy (including no immunomodulator or salicylate therapy).

Details on Dual Biologic Use

Patients who received dual biologic therapy (vedolizumab plus another biologic agent) at 14-weeks, 26-weeks, or 1-year did not demonstrate a significant difference in clinical remission or corticosteroid free remission rates ($p>0.1$) compared with patients on vedolizumab monotherapy. However, IBD patients not on dual biologic agents at 2-years were more likely to be in corticosteroid free clinical remission compared with IBD patients requiring vedolizumab plus another biologic agent ($p=0.004$). Combination regimens are further described in Table 2.

Of the 8 patients with CD who continued to receive another biologic agent during the induction of vedolizumab, 13% (1/8) achieved clinical remission at week 14 but 75% (6/8) continued to have mild disease activity. The one patient who achieved clinical remission at week 14 was on ustekinumab concomitantly during induction and maintenance but ultimately was taken off vedolizumab before reaching the 1-year timepoint. Furthermore, of the mild disease activity group, 1 patient ultimately achieved clinical remission by 26-weeks and remained on vedolizumab monotherapy by 1-year and 2-years post vedolizumab initiation.

Of the 5 patients with UC who continued to receive an anti-TNF agent during the induction of vedolizumab, 60% (3/5) achieved corticosteroid free clinical remission at week 14. Two of the patients were on adalimumab during induction and remained on adalimumab through

26-weeks and 1-year but thereafter developed mild disease activity despite the dual biologic therapy. Furthermore, the remaining patient who had achieved corticosteroid free clinical remission at week 14 was on infliximab during induction but was taken off of infliximab at week 14. This patient continued to remain in corticosteroid free clinical remission at the 2-year timepoint on vedolizumab monotherapy.

A second biologic agent was not added for any patients with UC who were initiated on vedolizumab monotherapy. However, 3 patients with CD who had undergone standard induction with vedolizumab alone required the addition of a second biologic agent at or after 1 year of the monotherapy. One of these patients received infliximab but ultimately stopped vedolizumab therapy. The other 2 patients received ustekinumab in addition to vedolizumab and ultimately only one of the two patients was able to remain on dual ustekinumab/vedolizumab therapy at 2 years but with continued mild disease activity.

Corticosteroid free remission rates in patients with colonic only disease (including UC and colonic only CD) was no different compared with patients with SB involvement (ileocolonic CD) at both 26-week (colonic only 50% versus ileocolonic 50%) and 1-year (colonic only 58% versus ileocolonic 50%) timepoints. Clinical remission or corticosteroid plus other biologic agent free remission rates also did not differ.

No significant ($p>0.1$) difference was found relating to gender in any of the outcomes examined.

Durability of Vedolizumab

At week 26, 76% (13/17) of patients with CD and 74% (14/19) with UC remained on therapy (Figures 1 and 2). Of the patients with 52-week outcomes available, 65% (11/17) with

CD and 68% (13/19) with UC remained on vedolizumab. By 2-years, 9 patients with CD and 8 patients with UC had discontinued vedolizumab therapy due to severity of disease. No significant ($p>0.05$) difference between CD or UC was found in any of these outcomes.

Among the patients remaining on vedolizumab at 1-year, 64% of CD patients (4-week interval n=3, 6-week interval n= 4) and 85% of UC patients (4-week interval n= 6, 6-week interval n= 5) were on an intensified regimen compared with the adult conventional dosing of every 8-week infusions (Supplementary Figure 2). This ratio of intensified treatment increased to 80% of CD (4-week n=3, 6-week n=1) and 100% of UC patients (4-week n=5, 6-week n=2) by 2-years. No significant ($p>0.05$) difference between standard versus intensified dosing regimens was observed in any of these outcomes.

Surgical Outcomes

Seven patients with UC (35%) and 2 patients with CD (10%) required surgical intervention (partial/total colectomy or diverting ileostomy) following initiation of vedolizumab. The time from initiation of vedolizumab to surgical intervention varied from 3 months to 26 months (median time of 14 months). Among all patients who required surgical intervention, 3 patients were in clinical remission by week 14, of which 2 patients also met criteria for corticosteroid free remission. However, by 1-year, only 2 patients remained in corticosteroid free remission. Four patients required surgical intervention in less than 1 year from the start of vedolizumab. All four of these patients were noted to have mild to moderate disease activity at week 14 and 75% (3/4) of the patients subsequently had discontinued vedolizumab before reaching week 26. The remaining patient had undergone a diverting ileostomy at 12-weeks

following initiation of vedolizumab but was able to enter clinical remission by 26-weeks and continued to remain in corticosteroid free remission at the 1-year timepoint.

Therapeutic Drug Monitoring

Twenty 20 patients (51%) (9 CD and 11 UC) had therapeutic drug monitoring (TDM: vedolizumab levels and antibodies). TDM timing was not standardized and the decision to obtain vedolizumab levels and antibodies was directed by the individual physicians. None of these patients developed antibodies to vedolizumab. Meaningful analyses could not be performed on vedolizumab TDM in this cohort due to the inconsistency in the timing.

Patient safety

No serious adverse reactions to vedolizumab during the observation period were reported. One mild, possible drug related event was reported in a patient who developed nausea and vomiting immediately following the 4th dose and was thereby discontinued from further therapy of vedolizumab.

Discussion

We report the largest real-life PIBD cohort treated with vedolizumab with 1-year and 2-year outcomes to date. Response to vedolizumab or the effectiveness of this biologic decreased over time. Other pediatric studies generally report similar findings, but have not examined 2-year outcomes.[8,12-15] In the 2017 study by Ledder, *et al.*, at week 14, 25% of CD patients (n=16) and 47% of UC patients (n=34) were in corticosteroid free clinical remission. At week 22, 36% of CD (n =14) and 46% of UC patients (n=26) were in clinical remission. This study also reported

1-year data with 25% (1/4) of CD patients and 60% (6/10) of UC patients in clinical remission and ultimately noted that vedolizumab was effective, especially in UC, in inducing and maintaining remission during long-term use.[13] UC specific efficacy of vedolizumab did not reach statistical significance compared with CD (p=0.56). Our study supports this latter result, since no difference in outcomes between patients with CD and UC patients was observed. This finding underscores the importance of independent, larger cohort examinations of biologic effectiveness in PIBD. Our results also reflect adult trial outcomes, which report 32% of CD patients and 39% of UC patients in corticosteroid free clinical remission at 1-year, although only 16% of our UC patients were in clinical remission at the same time point.[7] Our cohort also included several patients who received dual biologic therapy from the initiation or during the maintenance phases. Noteworthy, patients who were on dual biologic agents at 2-years were less likely to be in clinical remission compared with patients on single biologic therapy (p=0.004). This alludes to the disease severity of our cohort. As reported by others, we did not find any serious adverse reaction to vedolizumab.

Most of our patients who were maintained on vedolizumab past 1-year and 2-year timepoints required interval intensification. All patients with UC at 2-years were on an intensified regimen. These findings suggest that intensified regimens may support the long-term maintenance of vedolizumab therapy. Intensified therapy of biologic agents in PIBD is becoming increasingly more common compared with standard adult practices.[18] *Jongsma et al.*, recently reported that younger pediatric patients on infliximab were more likely to be on intensified therapy to maintain clinical remission at 1-year.[20] Similar results have been observed for other biologics such as adalimumab[21] and ustekinumab[22] in the pediatric population. Our study also favors the use of intensified therapy for maintenance of vedolizumab in PIBD.

With increasing availability and ease of testing, TDM for other biologic agents such as adalimumab and infliximab in pediatric patients is becoming more common to optimize treatment and potentially clinical outcomes.[23] TDM of vedolizumab has been indicated as a useful tool in adult patients.[24] However, in the pediatric population, data on TDM for vedolizumab are limited to one study recently published by Aardoom, *et al*. who concluded that patients with CD may benefit from routine TDM and intensified dosing regimens.[25] The limited data on vedolizumab TDM in the pediatric population is likely due to the lack of standardized level testing in clinical practice. Prospective studies on pediatric pharmacokinetics for vedolizumab such as reported recently by Hyams *et al.* will further our understanding on optimized use of vedolizumab and TDM in PIBD.[16]

Although the regional/single center nature of this work may be considered a limitation, Shiau *et al.*[26] have suggested that consistency of medical care in single centers may improve the accuracy of clinical studies in IBD. A recent study from the largest prospective cohort on PIBD patients has underscored the significant variation in clinical care (including diagnosis and treatment) among the North American medical centers involved.[27] This work supports our premise on single center studies potentially providing higher accuracy when examining questions on management in PIBD even with smaller sample sizes than in multi-center cohorts. Regardless, our single center study also calls for standardized approaches with respect to TDM (by highlighting the lack thereof in real-life practice at a single center).

This study is limited by its retrospective nature and therefore in the ability to control for treatment regimens and for testing and follow up. PUCAI scores were used in all patients, including those with CD, due to limited data available and inability to calculate CD specific scores for disease activity (PCDAI). Although the largest single center cohort of its kind, this

work is limited by its cohort size and the lack of standardized measures for TDM use as highlighted above. Furthermore, our study included CD patients with only ileocolonic or colonic predominant disease. Therefore, our data cannot be extrapolated for CD patients with small bowel only or upper gastrointestinal disease.

Our observations indicate that vedolizumab is safe, but its overall efficacy declines with time in anti-TNF exposed CD and UC patients. Our findings also favor the need for intensified treatment regimens of vedolizumab in PIBD to promote long term maintenance of therapy. These findings emphasize the need for prospective optimization of treatment with vedolizumab and the ongoing requirement for novel preventative and therapeutic measures to combat this highly morbid disease group.

References:

1. Nasiri S, Kuenzig ME, and Benchimol, E. Long-term outcomes of pediatric inflammatory bowel disease. *Semin Pediatr Surg* 2017;26:398-404.
2. Ng S, Shi H, Hamidi N, *et al.* Worldwide incidence and prevalence of inflammatory bowel disease in the 21st century: a systematic review of population-based studies. *Lancet* 2018;390:2769-2778.
3. Sykora J, Pomahacova R, Kreslova M, *et al*. Current global trends in the incidence of pediatric-onset inflammatory bowel disease. *World J Gastroenterol* 2018;24:2741-2763.
4. Sonavane AD, Sonawane P, and Amarapurkar DN. Inflammatory bowel disease across the age continuum: similarity and disparity. *Indian J Pediatr* 2018;85:989-994.
5. Assa A, Bronsky J, Kolho KL, *et al*. Anti-TNF[alpha] treatment after surgical resection for Crohn's Disease is effective despite previous pharmacodynamics failure. *Inflamm Bowel Dis* 2017;23:791-797.
6. Ihekweazu F, Fofanova T, Palacios R, *et al.* Progression to colectomy in the era of biologics: A single center experience with pediatric ulcerative colitis. *J Pediatr Surg* 2020;55:1815-1823.
7. Engel T, Ungar B, Yung DE, *et al*. Vedolizumab in IBD – Lessons from real-world experience; a systematic review and pooled analysis. *J Crohn's Colitis* 2018;12:245-257.
8. Singh N, Rabizadeh S, Jossen J, *et al*. Multi-center experience of vedolizumab effectiveness in pediatric inflammatory bowel disease. *Inflamm Bowel Dis* 2016;22:2121-2126.
9. Feagan BG, Rutgeerts P, Sands BE, *et al*. Vedolizumab as induction and maintenance therapy for ulcerative colitis. *N Engl J Med* 2013;369:699-710.

10. Sandborn WJ, Feagan BG, Rutgeerts P, *et al*. Vedolizumab as induction and maintenance therapy for Crohn's disease. *N Engl J Med* 2013;369:711-721.

11. Lenti MV, Levison S, Eliadou E, *et al*. A real-world, long-term experience on effectiveness and safety of vedolizumab in adult patients with inflammatory bowel disease: The Cross Pennine study. *Dig Liver Dis* 2018;50:1299-1304.

12. Conrad MA, Stein RE, Maxwell EC, *et al*. Vedolizumab therapy in severe pediatric inflammatory bowel disease. *Inflamm Bowel Dis* 2016;22:2425-2431.

13. Ledder O, Assa A, Levine A, *et al*. Vedolizumab in paediatric inflammatory bowel disease: a retrospective multi-centre experience from the paediatric IBD Porto group of ESPGHAN. *J Crohn's Colitis* 2017;11:1230-1237.

14. Schneider A, Weghuber D, Hetzer B, *et al*. Vedolizumab use after failure of TNF-∝ antagonists in children and adolescents with inflammatory bowel disease. *BMC Gastroenterol* 2018;18:140.

15. Hajjat TM, Mosha M, Whaley KG, *et al.* Vedolizumab experience in children and adolescents with inflammatory bowel disease: a multicenter observational study. *Crohns & Colitis 360.* 2021;3:1-8.

16. Hyams JS, Turner D, Cohen SA, *et al.* Pharmacokinetics, safety, and efficacy of intravenous vedolizumab in paediatric patients with ulcerative colitis or Crohn's disease: results from the phase 2 HUBBLE study. *J Crohns Colitis.* 2022. doi: 10.1093/ecco-jcc/jjac036. [Epub ahead of print].

17. Breton J, Kastl A, Hoffman N, *et al.* Efficacy of combination antibiotic therapy for refractory pediatric inflammatory bowel disease. *Inflamm Bowel Dis.* 2019;25:1586-1593.

18. Turner D, Otley AR, Mack D, *et al.* Development, validation, and evaluation of a pediatric ulcerative colitis activity index: a prospective multicenter study. *Gastroenterology.* 2007;133:423–432.

19. Assa A, Rinawi F, and Shamir R. The long-term predictive properties of the Paris classification in pediatric inflammatory bowel disease patients. *J Crohn's Colitis* 2018;12:39-47.

20. Jongsma M, Winter DA, Huynh HQ, *et al.* Infliximab in young paediatric IBD patients: it is all about the dosing. *Eur J Pediatr* 2020;179:1935-1944.

21. Croft NM, Faubion Jr WA, Kugathasan S, *et al.* Efficacy and safety of adalimumab in paediatric patients with moderate-to-severe ulcerative colitis (ENVISION I): a randomised, controlled, phase 3 study. *Lancet Gastroenterol Hepatol* 2021;6:616-627.

22. Do P, Andersen J, Patel A, *et al.* Augmented ustekinumab dosing is needed to achieve clinical response in patients with anti-TNF refractory pediatric Crohn's disease: a retrospective chart review. *F1000Res* 2020;9:316.

23. Carman N, Mack DR, and Benchimol EI. Therapeutic drug monitoring in pediatric inflammatory bowel disease. *Curr Gastroenterol Rep* 2018;20:18.

24. Ward MG, Sparrow MP and Roblin X. Therapeutic drug monitoring of vedolizumab in inflammatory bowel disease: current data and future directions. *Therap Adv Gastroenterol* 2018;11:1-10.

25. Aardoom A, Jongsma M, de Vries A, *et al.* Vedolizumab trough levels in children with anti-TNF refractory inflammatory bowel disease. *J Pediatr Gastroenterol Nutr* 2020;71:501-507.

26. Shiau H, Ihekweazu FD, Amin M, *et al*. The unique inflammatory bowel disease phenotype of pediatric primary sclerosing cholangitis: a single center study. *J Pediatr Gastroenterol Nutr* 2017;65:404-409.

27. Krishnakumar C, Ballengee CR, Liu C, *et al*. Variation in care in the management of children with Crohn's disease: data from a multicenter inception cohort study. *Inflamm Bowel Dis* 2019;25:1208-1217.

Figure Legend

Figure 1: Disease activity and clinical outcomes in patients with Crohn's disease on vedolizumab treatment at 14-weeks, 26-weeks, 1-year and 2-years.

Figure 2: Disease activity and clinical outcomes in patients with ulcerative colitis on vedolizumab treatment at 14-weeks, 26-weeks, 1-year and 2-years.

Figure 1

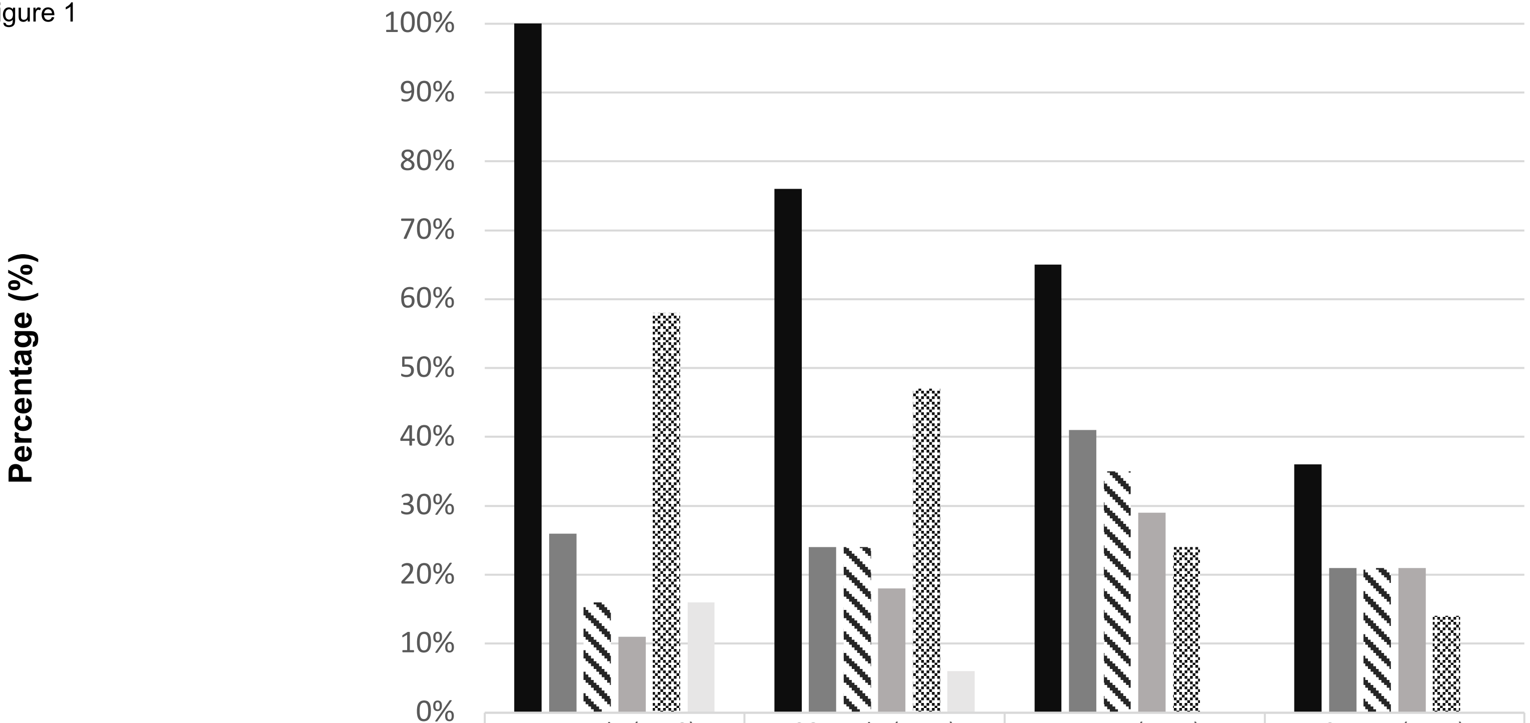

	14-weeks (n=19)	26-weeks (n=17)	1-year (n=17)	2-years (n=14)
CD Patients Remaining on Therapy	100%	76%	65%	36%
Clinical Remission	26%	24%	41%	21%
Corticosteroid Free Clinical Remission	16%	24%	35%	21%
Corticosteroid and Other Biologic Agent Free Clinical Remission	11%	18%	29%	21%
Mild Disease Activity	58%	47%	24%	14%
Moderate/Severe Disease Activity	16%	6%	0%	0%

Figure 2

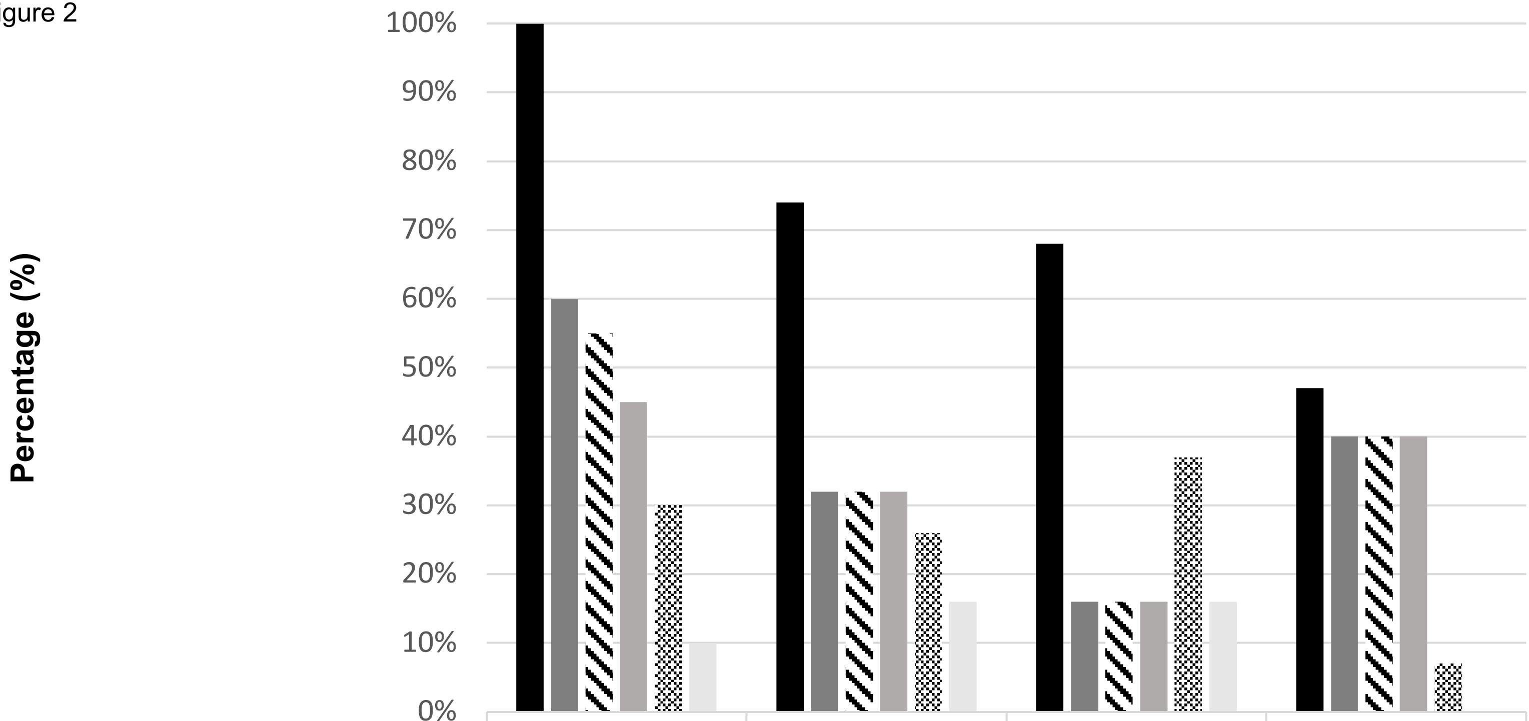

	14-weeks (n=20)	26-weeks (n=19)	1-year (n=19)	2-years (n=15)
UC Patients Remaining on Therapy	100%	74%	68%	47%
Clinical Remission	60%	32%	16%	40%
Corticosteroid Free Clinical Remission	55%	32%	16%	40%
Corticosteroid and Other Biologic Agent Free Clinical Remission	45%	32%	16%	40%
Mild Disease Activity	30%	26%	37%	7%
Moderate/Severe Disease Activity	10%	16%	16%	0%

Table 1: Baseline patient characteristics at start of vedolizumab therapy

	Total [n=39]	CD [n=19]	UC [n=20]
Male	19 [49%]	9 [47%]	10 [50%]
Ethnicity			
Hispanic	9 [23%]	3 [16%]	6 [30%]
Non-Hispanic	30 [77%]	16 [84%]	14 [70%]
Age at vedolizumab initiation year, mean (range)	14.5 [5-19]	14.3 [6-19]	14.8 [5-19]
Disease duration months, mean (range)	52.3 [3-201]	70.2 [10-201]	35.4 [3-143]
Number of previous biologic agents, n(%)			
0	1 [3%]	1 [5%]	0 [0%]
1	22 [56%]	9 [47%]	13 [65%]
2	16 [41%]	9 [47%]	7 [35%]
Previous biologic agents, n(%)			
Infliximab	33 [85%]	13 [68%]	20 [100%]
Adalimumab	19 [49%]	12 [63%]	7 [35%]
Certolizumab	1 [3%]	1 [5%]	0 [0%]
Ustekinumab	1 [3%]	1 [5%]	0 [0%]
Reason for discontinuation, n(%)			
Infliximab			
Primary Non-Responder	11 [33%]	2 [15%]	9 [45%]
Loss of Response	12 [36%]	6 [46%]	6 [30%]
Adverse Reaction	9 [27%]	4 [31%]	5 [25%]
Other	1 [3%]	1 [8%]	0 [0%]
Adalimumab			
Primary Non-Responder	4 [21%]	1 [8%]	3 [43%]
Loss of Response	15 [79%]	11 [92%]	4 [57%]
Certolizumab			
Loss of Response	1[100%]	1 [100%]	0 [0%]
Ustekinumab			

Primary Non-Responder	1[100%]	1 [100%]	0 [0%]
Behavior phenotype (CD), n(%)			
Nonstricturing, nonpenetrating [B1]	-	12 [63%]	-
Stricturing [B2]	-	1 [5%]	-
Penetrating [B3]	-	1 [5%]	-
Both structuring and penetrating [B2B3]		5 [26%]	
Lower gastrointestinal involvement (CD), n(%)			
Terminal ileum only [L1]	-	0 [0%]	-
Colonic only [L2]	-	7 [37%]	-
Ileocolonic [L3]	-	12 [63%]	-
Upper gastrointestinal involvement (CD), n(%)	-	15 [79%]	-
Perianal involvement (CD), n (%)	-	7 [37%]	-
Behavior phenotype (UC), n(%)			
Ulcerative proctitis [E1]	-	-	0 [0%]
Left-sided UC [E2]	-	-	0 [0%]
Extensive [E3]	-	-	1 [5%]
Pancolitis [E4]	-	-	19 [95%]
Severity (UC), n(%)			
Never severe [S0]	-	-	0 [0%]
Ever severe [S1]	-	-	20 [100%]

Table 2: Combination therapeutic agents applied with vedolizumab in the patients who were maintained on this biologic.

	14-Weeks	26-Weeks	1-Year	2-Years
CD	*n=19*	*n=13*	*n=11*	*n=5*
Corticosteroids	8 [42%]	2 [15%]	2 [18%]	0 [0%]
Other Biologic Agents	7 [37%]	4 [31%]	4 [36%]	2 [40%]
Other Immunomodulators	4 [21%]	3 [23%]	3 [27%]	1 [20%]
UC	*n=20*	*n=14*	*n=13*	*n=7*
Corticosteroids	5 [25%]	2 [14%]	0 [0%]	0 [0%]
Other Biologic Agents	3 [15%]	2 [14%]	2 [15%]	1 [14%]
Other Immunomodulators	7 [35%]	4 [29%]	3 [23%]	2 [29%]

Click here to access/download
Supplemental Data File (doc, pdf, etc.)
Supplementary Data September 2022.docx

Wolters Kluwer

JPGN Reports - Open Access License Agreement

This OPEN ACCESS LICENSE AGREEMENT (this "Agreement"), dated as of.

10/26/22

DATE

(the "Effective Date"), by and between Wolters Kluwer Health, Inc., operating as Medical Research / Lippincott Williams & Wilkins, a Delaware corporation, having its principal place of business at Two Commerce Square, 2001 Market Street, Philadelphia, PA 19103 (the "Publisher"), and the corresponding author listed on Schedule A to this Agreement (the "Author", and together with the Publisher, the "Parties").

1. Grant of License

The Author hereby grants to the Publisher and its Affiliates the exclusive, worldwide, royalty free, perpetual (for the duration of the applicable copyright) right and license to use the Work for all commercial or educational purposes, including, but not limited to, publishing, reproducing, marketing, distributing (themselves and through distributors), sublicensing, and selling copies of the Work throughout the world for the Term. If the Author is a United States government employee, such license grant shall be limited to the extent the Author is able to grant such license.

2. Warranties, Indemnification, and Limitation of Liability

a. The Author represents and warrants that:

(i) it has the right and power to enter into this Agreement, to grant the rights and licenses granted pursuant to this Agreement, and to perform all of its other obligations contained in this Agreement;

(ii) it has not previously assigned, transferred or otherwise encumbered the rights or licenses granted pursuant to this Agreement; and that the person executing this Agreement on the Author's behalf is authorized to do so;

(iii) the Work and the licenses granted herein do not and will not infringe upon, violate or misappropriate any intellectual property rights or any other proprietary right, contract or other right or interest of any third party;

(iv) if the Work is a multi-authored Work, the Author has obtained written permission from each author of the Work to enter into this Agreement on behalf such author, and each such author has read, understands and has agreed to the terms of this Agreement; and

(v) the Author has obtained any necessary releases and permissions to quote from other sources in the Work and to include any works and materials in the Work and all such releases and permissions are in full force and effect.

b. The Author hereby indemnifies the Publisher and its directors, officers, employees, agents, and representatives and agrees to defend and hold them harmless from and against any and all liability, damage, loss, costs or expenses (including reasonable attorney's fees and costs of settlement) incurred by any such party arising out of, or relating to any misrepresentation in, or breach or alleged breach of the Author's representations or warranties in this Agreement. If the Author fails to promptly or diligently pursue any defense of any indemnified party, the indemnified parties, or any of them, may assume such defense at the Author's expense. The obligations of this indemnification will survive any termination or expiration of this Agreement.

c. The Publisher represents and warrants that it has the right and power to enter into this Agreement and to perform its obligations contained in this Agreement, and that the person executing this Agreement on the Publisher's behalf is authorized to do so.

d. The Publisher hereby indemnifies the Author and agrees to defend and hold the Author harmless from and against any and all liability, damage, loss, costs or expenses

(including reasonable attorney's fees and costs of settlement) incurred by the Author arising out of, or relating to any misrepresentation in, or breach or alleged breach of the Publisher's representations or warranties in this Agreement. If the Publisher fails to promptly or diligently pursue any defense of the Author, the Author may assume such defense at the Publisher's expense. The obligations of this indemnification will survive any termination or expiration of this Agreement.

e. EXCEPT AS OTHERWISE SET FORTH IN THIS AGREEMENT, NEITHER PARTY MAKES ANY OTHER, AND HEREBY DISCLAIMS ALL OTHER, REPRESENTATIONS AND WARRANTIES OF ANY KIND, WHETHER EXPRESS, IMPLIED, STATUTORY OR OTHERWISE, INCLUDING, WITHOUT LIMITATION, WARRANTIES OF TITLE, MERCHANTABILITY, FITNESS FOR A PARTICULAR PURPOSE, NONINFRINGEMENT, OR THE ABSENCE OF LATENT OR OTHER DEFECTS, ACCURACY, OR THE PRESENCE OR ABSENCE OF ERRORS, WHETHER OR NOT DISCOVERABLE.

f. EXCEPT TO THE EXTENT REQUIRED BY APPLICABLE LAW, IN NO EVENT WILL EITHER PARTY BE LIABLE TO THE OTHER PARTY BASED UPON ANY LEGAL THEORY FOR ANY SPECIAL, INCIDENTAL, CONSEQUENTIAL, PUNITIVE, OR EXEMPLARY DAMAGES ARISING OUT OF THIS LICENSE OR THE USE OF THE WORK, EVEN IF A PARTY HAS BEEN ADVISED OF THE POSSIBILITY OF SUCH DAMAGES.

3. Creative Commons License.

Creative Commons Licenses are subject to items selected in item 1, 2 and 3 in the Schedule B.

a. CCBY-NC-ND – NonCommercial-NonDerivitives Creative Commons License

The Author acknowledges and agrees that the Work will be published by the Publisher in (the "Journal") and made freely available to users under the terms of the Attribution-NonCommercial-NoDerivs 4.0 Creative Commons License, as currently displayed at http://creativecommons.org/licenses/by-nc-nd/4.0/legalcode (the "CC BY-NC-ND"). The Author acknowledges and agrees that that Publisher is the exclusive "Licensor", as defined in the CC BY-NC-ND, of the Work and that the Publisher may make the Work freely available to all users under the terms of the CC BY-NC-ND.

b. CC BY – Creative Commons License

The Author acknowledges and agrees that the Work will be published by the Publisher in (the "Journal") and made freely available to users under the terms of the Creative Commons Attribution- 4.0
Creative Commons License, as currently displayed at http://creativecommons.org/licenses/by-nc-sa/4.0/legalcode (the "CC BY"). The Author acknowledges and agrees that the Publisher is the exclusive "Licensor", as defined in the CC BY, of the Work and that the Publisher may make the Work freely available to all users under the terms of the CC BY.

4. Royalties.

The Author acknowledges and agrees that this Agreement entitles the Author to no royalties or fees. To the maximum extent permitted by law, the Author waives any and all rights the Author may have to collect royalties or other fees in relation to the Work or in respect of any use of the Work by the Publisher or its sublicensees.

5. Miscellaneous.

a. Assignment. This Agreement may not be assigned or transferred, in whole or in part, by either party without the prior written consent of the other party. Notwithstanding the above, the Publisher may assign this Agreement without the written consent of the Author (i) to an entity succeeding, whether by sale, merger or other corporate reorganization, to substantially all of the Publisher's assets and business activity, or (ii) to a corporation or organization that obtains the right to publish the Journal from the Publisher. The Publisher may assign this Agreement to any of its affiliates. This Agreement will be binding upon and inure to the benefit of the parties hereto and their respective successors and permitted assigns.

b. Counterparts. This Agreement may be executed in two or more counterparts, each of which shall be deemed an original, but all of which together shall constitute one and the same document. Facsimile or Portable Document Format (PDF) signatures will be deemed original signatures for purposes of this Agreement.

c. Entire Agreement; Amendment. This Agreement sets forth the entire agreement of the parties on the subject hereof and supersedes all previous or contemporaneous oral or written representations or agreements relating to the rights and duties provided herein, and may not be modified or amended except by written agreement of the parties.

d. Force Majeure. Neither party shall be liable for any default or delay on its part in performing any obligation under this Agreement if such default or delay is caused by natural disaster, accident, war, civil disorder, strike or any other cause beyond the reasonable control of such party. In the event that either party is prevented by such an occurrence or circumstance for a period of more than ninety (90) days from fulfilling its obligations under this Agreement, the other party may terminate this Agreement upon thirty (30) days' written notice.

e. Governing Law. This Agreement shall be governed in all respects according to the laws of the State of New York without giving effect to the principles of conflict of law thereof.

f. Headings. All headings are for reference purposes only and shall not affect the meaning or interpretation of any provision hereof.

g. Severability. If any provision of this Agreement is held to be illegal, invalid, or unenforceable under the present or future laws, then such provision shall be revised by a court of competent jurisdiction to be enforceable if permitted under applicable law, and otherwise shall be fully severable. In any event, this Agreement shall be construed and enforced as if such illegal, invalid, or unenforceable provision had never comprised a part of this Agreement, and the remaining provisions of this Agreement shall remain in full force and effect and shall not be affected by the illegal, invalid, or unenforceable provision or by its severance from this Agreement.

h. Status of the Parties. The parties are independent contractors. Nothing in this Agreement is intended to or shall be construed to constitute or establish any agency, joint venture, partnership or fiduciary relationship between the parties, and neither party has the right or authority to bind the other party nor shall either party be responsible for the acts or omissions of the other.

i. Waiver; Amendment. The waiver by either party of or the failure by either party to claim a breach of any provision of this Agreement shall not be, or be held to be, a waiver of any subsequent breach or affect in any way the further effectiveness of any such provision. No term or condition of this Agreement may be waived except by an agreement by the parties in writing.

j. Waiver of Jury Trial. EACH PARTY HEREBY WAIVES ITS RIGHT TO A JURY TRIAL IN CONNECTION WITH ANY DISPUTE OR LEGAL PROCEEDING ARISING OUT OF THIS AGREEMENT OR THE SUBJECT MATTER HEREOF.

[Signature Page Follows]

Schedule A

This Schedule A must be completed by Author in its entirety. The Publisher is unable to publish the Work unless this Schedule A is completely filled out.

JPGNREP-21-210R3
Article Tracking #

A Single Center Study of Long-Term Effectiveness of Vedolizumab in anti-TNF refractory Pediatric Inflammatory Bowel Disease
Article Title (the "Work")

Halee Patel
Corresponding Author Name (the "Author")

Wolters Kluwer
Copyright Owner's Name

JPGN Reports
Name of Journal in which Work is to be Published

Schedule B - CREATIVE COMMONS LICENSE

Please select the Creative Commons License pursuant to which the Publisher will license the Work (a description of each license, and the license terms, can be reviewed at http://www.creativecommons.org/licenses):

Select one from the list below:

☑ **Attribution CC BY**. Creative Commons describes this license as follows: "This license lets others distribute, remix, tweak, and build upon your work, even commercially, as long as they credit you for the original creation. This is the most accommodating of licenses offered. Recommended for maximum dissemination and use of licensed materials."

☐ **Attribution-NonCommercial-NoDerivs CC BY-NC-ND.** Creative Commons describes this license as follows: "This license is the most restrictive of our six main licenses, only allowing others to download your works and share them with others as long as they credit you, but they can't change them in any way or use them commercially."

☐ **US Government Employee.** If the Work or a portion of it has been created in the course of any author's employment by the United States Government, check the "Government" box at the end of this form. A work prepared by a government employee as part of his or her official duties is called a "work of the U.S. Government" and is not subject to copyright. If it is not prepared as part of the employee's official duties, it may be subject to copyright.

If "Government" is chosen, please do not choose a Copyright Clearance License. The work will be published with "Written work prepared by employees of the Federal Government as part of their official duties is, under the U.S. Copyright Act, a "work of the United States Government" for which copyright protection under Title 17 of the United States Code is not available. As such, copyright does not extend to the contributions of employees of the Federal Government."

SIGNATURE PAGE

The Corresponding Author acknowledges and agrees that the Corresponding Author is entering into, and has executed, the Agreement on behalf of the Corresponding Author and each other author named as contributing to the Article (each such author, an "Author", and collectively, the "Authors"). The Corresponding Author represents and warrants that the Corresponding Author has obtained permission from each Author to enter into the Agreement on behalf of such Author and the Corresponding Author and each Author has read, understands, and has agreed to the terms of the Agreement, including, without limitation, the terms contained in the Agreement with respect to authorized reuse of the Article.

IN WITNESS WHEREOF, the Author has executed this License, effective as of the Effective Date.

Halee Patel

PRINT NAME

[illegible]

SIGNATURE

Important Note: Once you electronically sign this form you will not be able to make any additional changes to it

To electronically sign this form, click the signature field above and provide the information requested in the dialog boxes.
